# Interleukin‐11 promotes lung adenocarcinoma tumourigenesis and immune evasion

**DOI:** 10.1002/ctm2.70374

**Published:** 2025-07-17

**Authors:** Cristina Cirauqui, Laura Ojeda, Itziar Otano, Irene Pazos, Alba Santos, Eva M. Garrido‐Martín, Patricia Yagüe, Javier Ramos‐Paradas, Sonia Molina‐Pinelo, Giovanna Roncador, José Luis Solórzano, M. Teresa Muñoz, Patricia Cozar, Patricia Plaza, Rocío Suárez, Marta Jiménez, Roberto Moreno, Arantxa Rosado, Pablo Gámez, Ricardo García‐Luján, Jon Zugazagoitia, E. Alejandro Sweet‐Cordero, Mariano Barbacid, Amancio Carnero, Irene Ferrer, Luis Paz‐Ares

**Affiliations:** ^1^ H12O‐CNIO Lung Cancer Clinical Research Unit Health Research Institute Hospital 12 de Octubre (imas12)/Spanish National Cancer Research Center (CNIO) Madrid Spain; ^2^ Spanish Center for Biomedical Research Network in Oncology (CIBERONC) Madrid Spain; ^3^ Targeted Therapies for Precision Oncology Group Health Research Institute Hospital 12 de Octubre (imas12) Madrid Spain; ^4^ Biology Department Autónoma University of Madrid Madrid Spain; ^5^ Department of Pathology Yale University School of Medicine New Haven Connecticut USA; ^6^ Oncoheematology and Genetic department Institute of Biomedicine of Seville (IBIS) (HUVR, CSIC, Universidad de Sevilla) Sevilla Spain; ^7^ Biotechnology Program Spanish National Cancer Research Center (CNIO) Madrid Spain; ^8^ Pathological Anatomy and Molecular Diagnosis Service MD Anderson Cancer Center Madrid Madrid Spain; ^9^ Thoracic Surgery Service Hospital 12 de octubre Madrid Spain; ^10^ Medical Oncology Department Hospital Universitario Doce de Octubre Madrid Spain; ^11^ Department of Pediatrics University of California San Francisco San Francisco California USA; ^12^ Experimental Oncology Group Spanish National Cancer Research Center (CNIO) Madrid Spain; ^13^ Medical School Universidad Complutense Madrid Spain

**Keywords:** lung adenocarcinoma, IL‐11/IL‐11RA axis, tobacco smoke, immunosuppressive tumour microenvironment, therapeutic target

## Abstract

**Rationale:**

Interleukin‐11 (IL‐11) has emerged as a significant player in tumourigenesis, with implications across various cancer types. However, its specific role in driving tumour progression in lung adenocarcinoma (LUAD) remains elusive. IL‐11's multifaceted impact on both tumour cells and the tumour microenvironment underscores its potential as a therapeutic target in LUAD. This study aims to unravel the involvement of IL‐11 in LUAD progression and its influence on the tumour microenvironment.

**Methods:**

Here, we used transcriptomic and digital spatial profiling analyses together with clinic data from two retrospective LUAD patient cohorts. LUAD cell lines genetically engineered to overexpress or to silence IL‐11 or its receptor (IL‐11RA) were used for in vitro functional analysis and for in vivo experiments. Additionally, we used three different in vivo models: patient‐derived xenografts (PDXs), tobacco‐exposed mice and genetically engineered mouse models. A neutralising monoclonal antibody against IL‐11RA was produced and tested.

**Results:**

Our findings revealed a pivotal role for IL‐11 in driving tumourigenesis across various mouse models, highlighting its capacity to modulate tumour immunity towards an immunosuppressive microenvironment. Moreover, we observed a correlation between IL‐11 expression and poorer patient outcomes in LUAD. Notably, therapeutic targeting of IL‐11RA with a neutralising antibody demonstrated significant anti‐tumour efficacy in a PDX model.

**Conclusion:**

The IL‐11/IL‐11RA axis emerges as a critical driver of LUAD tumourigenesis, exerting its effects through enhanced tumour cell proliferation and remodelling of the tumour microenvironment. Our study highlights the therapeutic potential of disrupting this axis, suggesting that patients exhibiting elevated IL‐11 levels may benefit from therapies targeting the IL‐11/IL‐11RA pathway.

## Graphical abstract

IL‐11 drives tumour progression and is linked to poor survival in lung adenocarcinoma patients, as well as an immunosuppressive tumour microenvironment. Antibody targeting of the IL‐11 receptor (IL‐11RA) shows significant anti‐tumour effects in a patient‐derived xenograft model highlighting IL‐11 as a compelling therapeutic target.

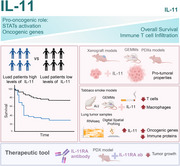

## 1 INTRODUCTION

More than 2 million new cases and 1.8 million deaths are reported annually due to lung cancer. This high number accounts for the highest cancer‐related fatalities globally. The high mortality rate is due to delayed detection and the restricted efficacy of available therapies. Tobacco smoke is linked to approximately 90% of cases.^1^ Non‐small cell lung cancer (NSCLC) comprise about 85% of all lung cancer diagnoses, with adenocarcinoma making up over half of these cases.^2^ The development of targeted therapies for several oncogenic driver genes and immunotherapies has contributed to a substantial reduction in mortality from NSCLC.^3^, ^4^ Although treatment with small‐molecule tyrosine kinase inhibitors is initially effective in lung cancer patients, acquisition of resistance almost inevitably occurs.^5^ Thus, more effective treatment strategies are urgently needed.

An intricate interplay of abnormally expressed cytokines, growth factors, chemokines and matrix‐modifying enzymes drives lung cancer development and accelerates its progression towards invasive metastasis.^6^, ^7^, ^8^ Tumour‐growth cytokines support cancer progression by activating autocrine signalling within tumour cells and facilitating paracrine interactions between these cells and their surrounding tumour microenvironment (TME).^6^ Clinical studies showed that increased concentrations of the interleukin (IL)‐6 family members are frequent in cancer and are often associated with poor clinical outcomes.^7^ IL‐11 is a member of the IL‐6 family of cytokines, involved in cancer progression across multiple cancer types such as colorectal cancer, gastric cancer, breast cancer, prostate cancer and endometrial cancer.^8^, ^9^, ^10^, ^11^, ^12^, ^13^, ^14^ A study by our group revealed that IL‐11 acts as a diagnostic biomarker for LUAD.^15^ IL‐11 signalling involves IL‐11 binding to the membrane bound receptor α (IL‐11RA), recruiting glycoprotein 130 (gp130). This leads to transphosphorylation and activation of JAK1, JAK2 and TYK2, converging on the phosphorylation of the transcription factor STAT3 and a transient activation of the RAS/ERK and PI3K pathways.^16^ Excessive activation of the IL‐11–STAT3 signalling pathway is considered a pathological mechanism of tumour growth and metastatic spread.^8^, ^17^, ^18^, ^19^ Despite the potential oncogenic role of the IL‐11 pathway reported in the context of NSCLC,^20^ the contribution of IL‐11 to LUAD progression and its implication in the TME are not well understood.

In this study, we demonstrate that the IL‐11 signalling pathway is an important player driving the progression of LUAD. We generated an IL‐11 pathway‐blocking antibody, observing its anti‐tumour impact in a PDX model. We further revealed that IL‐11 dysregulates the immunity of the tumour generating an immunosuppressive microenvironment in two murine models of tobacco–induced LUAD. These results were confirmed in tumour samples from LUAD patients. Here, we show that those patients with high IL‐11 cytokine expression levels have down‐regulated the expression of genes involved in the anti‐tumour immune response, whereas genes that participates in immune evasion and tumour progression are overexpressed.

## 2 MATERIALS AND METHODS

### 2.1 Clinical samples

Four cohorts of patients were used in this work: Cohort 1, 3 and 4 with samples from Hospital 12 de Octubre (H12O) in Madrid and Cohort 2 from The Cancer Genome Atlas (TCGA). TCGA information is based upon data generated by UCSC Xena. The cohort characteristics are summarised in Tables S1–S4. Samples of cohort 3 and 4 were selected from cohort 1. Resected tumour samples were fixed in formalin and embedded in paraffin in the case of cohort 1 and 4, while cohort 3 samples were fresh‐frozen with optimal cutting temperature (OCT) compound. For cohort 4, we selected tumour samples from cohort 1 that exhibited both the highest and the lowest levels of IL‐11 expression. For cohort 3, we selected tumour samples from cohort 1 that were available in OCT compound.

### 2.2 Mice

We selected TP57 and TP60 PDX models from our own collection of NSCLC PDX models. Resected lung tumours from early‐stage diagnosed patients were implanted subcutaneously and expanded in athymic nude mice.

Cell line‐derived xenografts were established in 6‐week‐old nude mice by inoculation with 1 × 10^6^ cells overexpressing either IL‐11 or IL‐11RA or empty vector (EV). Combinations of 1:1 cell variants were injected in PBS/Matrigel into both flanks. Tumour growth was measured twice weekly. The volume of the tumour was calculated using the formula: 0.5 × tumour length × tumour width^2^.

Three genetically engineered mice models (GEMM) of lung cancer were employed: *KRas^LSLG12Vge/+o^;P53^loxP/loxP^*, *KRas^LSLG12Vge/+o^;P53* ^(+/+)^ *IL‐11RA* WT and *KRas^LSLG12Vge/+o^;P53* ^(+/+)^ *IL‐11RA* KO. *KRas* ^LSLG12Vge/+o^;*P53* ^(+/+)^ *IL‐11RA* KO and *KRas* ^LSLG12Vge/+o^;*P53*(^+/+)^ *IL‐11RA* WT mice were generated by crossing *KRas* ^LSLG12Vge/+o^;*P53* ^(+/+)^ mice with *Il11ra1* ^tm1Wehi^ mice from The Jackson Laboratory. The *Il11ra1* ^tm1Wehi^ allele is a targeted null mutation in the *Il11ra1* gene, resulting in complete loss of IL‐11RA expression. Offspring were genotyped to identify animals either homozygous for the null allele KO (IL‐11RA KO) or wild‐type at the *Il11ra1* locus (IL‐11RA WT). Tumour size was measured by computed tomography (CT). For the tobacco‐exposure experiment, we employed 6‐week‐old A/J mice (RRID: IMSR_JAX:000646) from the Jackson Laboratory and a GEMM of lung cancer *KRas^LSLG12Vge/+o^;P53* ^(+/+)^.

### 2.3 Ethics statement/study approval

Written informed consent was provided by all patients for the collection, storage and use of donated samples. The described procedures were approved by the Ethics Committee of the H12O with Identification Numbers #16/364 #17/454.

All animal procedures received approval from the Ethical Committees of the Carlos III Health Institute and the Autonomous Community of Madrid (CAM) and were conducted following the International Guiding Principles for Biomedical Research Involving Animals, as established by the Council for International Organizations of Medical Sciences (Approval ID: PROEX 069/19, PROEX 307/19 and PROEX 239/19).

### 2.4 Cell lines

All tumour cell lines were purchased from ATCC, except for H1437 and H3122, which were provided by Dr Maina and Dr Koivunen, respectively. All cell lines were grown in accordance with ATCC indications and were validated and tested frequently for Mycoplasma.

### 2.5 Transfections

Cell lines were transfected as indicated in the instructions with TransIT‐X2 (Mirus, Madison, WI). IL‐11 (RC204493) and IL‐11RA (RC226571) cDNA clones were purchased from Origene (Rockville, MD) in the pCMV6 vector (PS1000001).

### 2.6 IL‐11 and IL‐11RA CRISPR/Cas9 gene targeting

sgRNAs for IL‐11 (TGACACTTGACTGGGCCGTG) and IL‐11RA (CAGTGTCCTGGTTTCGGGAT) were designed using the *Genetic Perturbation Platform* (http://www.broadinstitute.org/ARNi/public/analysis‐tools/sgARN‐design) and cloned in the plentiCRISPRv2. HEK293T cells were transfected with psPAX2 (plasmid #12260), pMD2.G (plasmid #12259) and plentiCRISPRv2 (plasmid #52961) plasmids from Addgene (Massachusetts, USA) to produce lentiviral particles.

### 2.7 Anti‐IL‐11RA monoclonal antibody production

An anti‐IL‐11RA monoclonal antibody was generated by the Monoclonal Antibody Unit at CNIO. The sequence encoding the extracellular domain of human IL‐11RA (NP_001136256, residues 1–365) was fused to a C‐terminal human IgG1 Fc sequence and a His‐tag in a CMV‐based pcDNA3 vector. Expi293 cells (ThermoFisher) were transfected with the pcDNA3–IL‐11RA–Fc–His plasmid using ExpiFectamine 293 Reagent (ThermoFisher) according to the manufacturer's protocol. IL‐11RA–Fc–His was purified from the culture supernatant using a HiTrap Protein A HP column fitted to an ÄKTA‐prime system (GE Healthcare).

Two Wistar rats (female, 6‐weeks‐old, RRID:RGD_2308816; Charles River Laboratories) were injected at 14‐day intervals 4 times intraperitoneally with 100 µg of IL‐11RA–Fc–His fusion protein and Freund's adjuvant Complete (Difco). The splenocytes were extracted and fused 3 days after a 150 µg last booster of the recombinant IL‐11RA–Fc–His protein, as described.^21^ Hybridoma supernatants were screened by enzyme‐linked immunosorbent assay (ELISA) and HEK‐293T cells were transfected with pCMV6–IL‐11RA–Myc–FLAG plasmid. The rat mAb that was raised against IL‐11RA (clone LAU490A, IgG2a) was cloned by the limiting dilution technique.

### 2.8 PDX‐derived organoids (PDXDO)

PDX tumours were minced into 1–2 mm fragments and processed with a digestion solution for 1–2 h at 37°C. Red Blood Cell lysis buffer (eBiosicences) was used to remove erythrocytes. 60,000 cells were embedded in 150 µL of Matrigel and seeded into 12‐well plates. Once Matrigel was solidified, complete human feeding media was added. For protein extraction, cells were incubated on ice for 30 min with cold Cell Recovery Solution (Corning). The pellet was washed twice and resuspended in protein extraction buffer (RIPA buffer [Sigma] with protease [cOmplete Mini without EDTA; Roche] and phosphatases [PhosSTOP EASYpack; Roche] inhibitors].

### 2.9 Surrogate assays

These assays were conducted as described in Guijarro et al.^22^ Growth curves, 15,000 cells/well were seeded in 12‐well plates, fixed every 2 days and assessed by crystal violet staining. The results were normalised to the day 0 plate. For soft agar assays and clonability, the number of colonies was counted after a period ranging from 2 weeks or 2 months after seeding. For the transwell migration assay, a total of 30 000 cells were seeded in serum‐free media on 8 µm pore polycarbonate transwell inserts (Costar‐Cultek) following the manufacturer's instructions. Cells were allowed to migrate for 24 h after the addition 0.5 mL of FBS‐containing conditioned medium to the lower chamber. Each assay was conducted at least three times, and three technical replicates were performed per condition.

### 2.10 rhIL‐11 in vitro stimulation

Cells lines or PDX‐derived organoids (PDXDO) were starved in FBS–free medium for 5 h and then stimulated with serum‐free medium containing 50 ng/mL recombinant human rhIL‐11 (Crystallography and Protein Engineering Unit, CNIO) for 15 min.

### 2.11 Neutralising antibody IL‐11RA treatment in vitro

250 000 cells from A549 cell line were seeded in 2.5 cm well plates or cells‐derived for PDX tumours were processed as is described in PDXDOs paragraph. Cells were incubated for 24 h at 37°C with 500 or 1000 µg/mL of neutralising antibody IL‐11RA and then were stimulated with 50 ng/mL of rhIL‐11 for 15 min.

### 2.12 Immunoblot

Western blot (WB) was performed as indicated by Quintanal‐Villalonga et al.^23^ with use of the antibodies described in Table S6. The bands were quantified with Image Lab software (Bio‐Rad). All values were normalised relative to β‐actin loading control.

### 2.13 Enzyme‐linked immunosorbent assay (ELISA)

IL‐11 levels were measured from cell culture supernatants, bronchoalveolar lavage fluids (BALFs) and tumours derived from GEMMs and PDXs, respectively, by ELISA (Quantiquine; R&D Systems) following the manufacturer's instructions. The secretion of IL‐11 was normalised to the total protein in each cell line measured by Bradford (BioRad). IL‐12p70 levels were measured in BALFs from A/J mice by ProcartaPlex Immunoassays (ThermoFisher) following the manufacturer's instructions.

### 2.14 RNA extraction and qRT‐PCR assay

For tumour samples from cohort 3, five additional sections (4 µm thick) of fresh‐frozen tissue in OCT (−80°C) were used for RNA extraction. This was carried out using the RNeasy Mini kit (#74104; QIAGEN, Hilden, Germany) following the manufacturer's instructions. For the quantification of RNA we used the Quantifluor RNA system (#E3310; Promega, Madrid, Spain), which provided a yield of 141.09 ng/µL in average and exceeded the requirements. Its quality was assessed with either the RNA 6000 Nano kit (#5067‐1511; Agilent, Santa Clara, CA, USA) or the RNA 6000 Pico kit (#5067‐1513; Agilent). All samples met quality standards for further analysis (according to RIN).

For qRT‐PCR assay, RNA samples were reverse transcribed with the TaqMan Reverse Transcription Kit (Life Technologies). To analyse the gene expression we used the TaqMan Master Mix Kit (Applied Biosystems) and TaqMan probes from Life Technologies: Hs00174148_m1 (IL‐11) and Hs99999907_m1 FAM (beta‐2‐microglobulin [β2M]). β2M expression was used to normalise the expression data.

### 2.15 Gene expression analysis

395 genes associated with the tumour–immune system communication were analysed for their expression with the Oncomine Immune Response Research Assay (OIRRA) (ThermoFisher Scientific, Waltham, MA, USA) as described in Ramos‐Paradas et al.^24^ Quality control measures and library preparation details are shown in Table S7, while raw and normalised gene expression data are available in Table S8.

### 2.16 In vivo treatments

PDX TP60 mice were treated with 500 µg/kg of rhIL‐11 (crystallography and protein engineering, CNIO) or vehicle (physiological serum) by intraperitoneal injection for 9 consecutive days. Mice were randomised into groups of six mice each for the control and IL‐11 treatment groups, with similar tumour sizes and SDs (200–400 mm^3^) for the two groups. Tumours were measured twice a week. At the end of treatment tumours were harvested.

In the GEMM *KRas^LSLG12Vge/+o^;P53^loxP/loxP^* mice, 500 µg/kg of rhIL‐11 (*N* = 6 mice) or vehicle (*N* = 7 mice) were administered by intraperitoneal injection three times/week for 4 weeks. Treatment started when tumour volume measured by micro‐CT achieved 3–5 mm^3^. At the end of the treatment, tumours were measured by micro‐CT and mice were sacrificed.

PDX TP57 mice received 300 µg/mice of IL‐11RA mAb, anti‐GST mAb and saline by intraperitoneal injection, three times/week, for 3 weeks. Mice were randomised into two groups: five mice for anti‐GST and six mice for anti‐IL‐11RA mAb, with similar tumour sizes and SDs (200–400 mm^3^) for the two groups. Tumours were measured twice a week, and mice were weighed once a week to monitor toxicity. Tumours were harvested at the end of treatment.

### 2.17 Tobacco smoke experiments

We used the same protocol described in Takahashi et al.^25^ with some modifications. Fresh smoke was diluted with filtered air and administered using a whole‐body exposure system (Cigarette Smoke Machine CSM‐SCSM, model CSESCM1). The average total suspended particulate matter measured at the nose ports was 10 mg/m^3^. For the A/J model, two groups of mice (five mice in each group) were administered the tobacco carcinogen, nitrosamine 4‐(methylnitrosamino)‐1‐(3‐pyridyl)‐1‐butanone (NNK). Male A/J mice were intraperitoneally injected with 70 mg/kg NNK (Toronto Research Chemicals) 1 week before initiation of MTS (mainstream tumour smoke) exposure. For the *KRas^LSLG12Vge/+o^;P53^(+/+)^ * mouse model, mice were intranasally inoculated with adeno‐Cre viruses to induce the development of LUAD tumours. In both models, for IL‐11 treatment, in the last smoking cycle, mice were treated with rhIL‐11 (500 µg/kg) intraperitoneally administered for three times/week for 3 weeks and the last day of the experiment. A third control group of mice (*n* = 5) was exposed to a normal air environment.

### 2.18 Immunohistochemistry

Samples were fixed in 10% formalin, embedded in paraffin blocks and cut with a microtome (Leica Biosystems). Patient tumour samples were prepared in H12O and slides were automatically stained in a BONDIII instrument (Leica Biosystems) using the reagents recommended by the supplier. For immunohistochemical staining of cell lines and PDX, an automatic platform (Window Discovery XT [Roche] or Austistainer Link [Dako]) was used in CNIO along with the manufacturer's reagents. The antibodies are described in Table S6. Results of the immunohistochemistry studies were evaluated by a clinical pathologist.

### 2.19 Flow cytometry

Single‐cell suspensions from lung tissues were prepared with a digestion solution (60 units/mL DNase1 + 70 units/mL Collagenase type 1 in HBBS) using a gentleMacs Dissociator. After lysing red blood cells (1× RBC Lysis Buffer; Invitrogen), cells were Fc‐blocked (BD Biosciences) for 15 min at room temperature. We used the LIVE/DEAD™ Fixable Aqua Dead Cell Stain (ThermoFisher) to discriminate dead cells. Samples were stained for extracellular markers listed in Table S6. Data were acquired on a BD LSRII Fortessa instrument and analysed using FlowJo v10. Specific gating strategies for analysis were defined in Figure S9.

### 2.20 Digital spatial profiling

A digital spatial profiling (DSP) protocol was carried out as described in Zugazagoitia et al.^26^ Differential expression proteins in the tumour compartment were evaluated between patients expressing high levels of IL‐11 compared with patients expressing low levels of IL‐11 using NanoString technologies. The immune‐oncology panel used is given in Table S5. Information about data from volcano plot is available in Table S9.

### 2.21 Statistics

We used the statistical software packages SPSS (IBM) and GraphPad Prism, with the appropriated statistical tests applied as indicated in each figure. Significant differences were indicated on figures as *<.05, **<.01 and ***<.001. Survival analysis was performed using the Kaplan–Meier method, with *p* values calculated via the Log‐Rank model. The Cox proportional hazards model was employed to assess hazard ratio (HR) values. Overall survival (OS) was defined as the period between diagnosis and the last clinical review or death.

## 3 RESULTS

### 3.1 Increased expression of IL‐11 is associated with poor overall survival in LUAD patients

**FIGURE 1:**
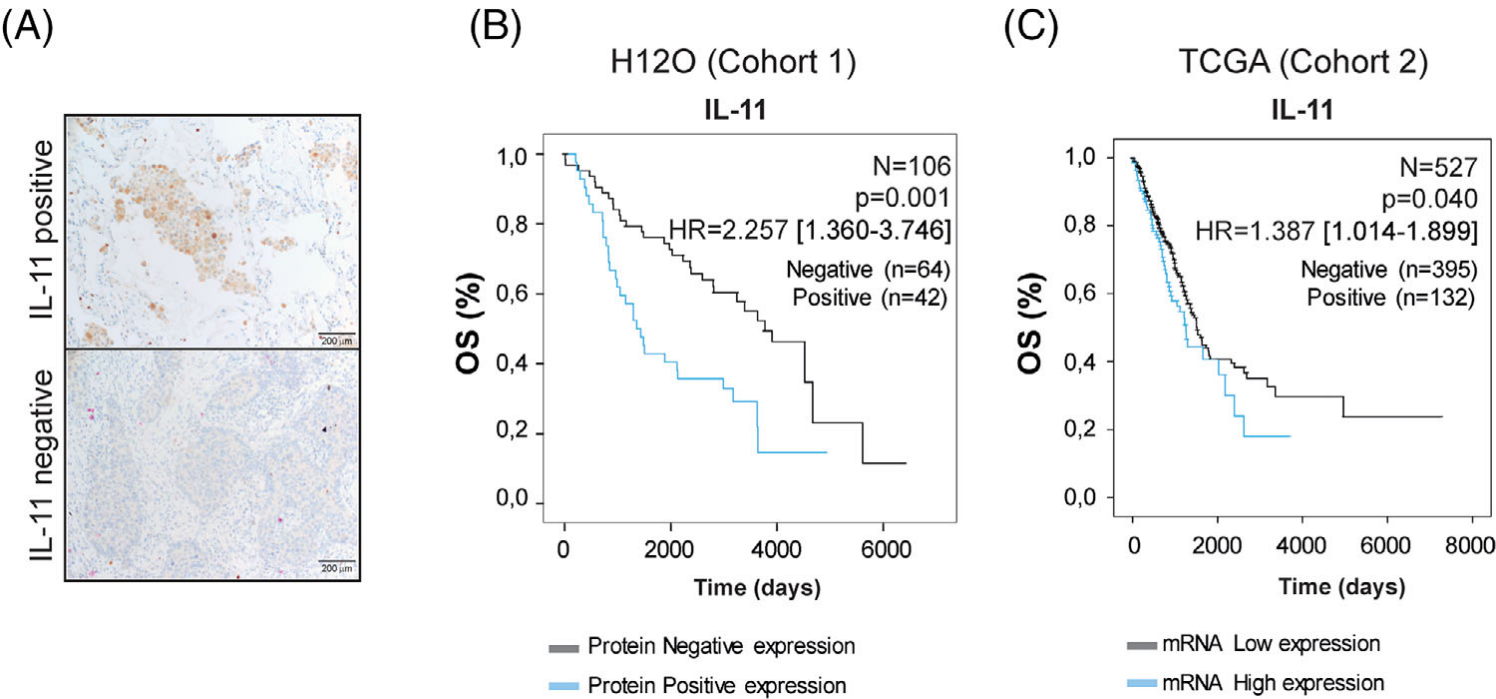
Augmented expression of IL‐11 is associated with poorer outcomes in LUAD patients. (A) Representative microphotographs of IL‐11 immunohistochemistry (IHC) in two independent lung adenocarcinoma patients. Scale bar 200 µm. (B) and (C) Kaplan–Meier curves of overall survival (OS) were plotted for the LUAD patients from: cohort 1; H12O *n* = 106 and cohort 2; TCGA *n* = 527, respectively. (B) For cohort 1, patient samples were divided in positive immunohistochemistry staining which corresponded to samples with weak, moderate or strong IL‐11 expression, and negative staining that included samples without any IL‐11 expression. (C) For cohort 2, LUAD patient samples were divided into high or low IL‐11 mRNA expression levels, establishing the third quartile as cut‐off. The Kaplan–Meier method (*p < .05, **p < .01) was used for survival analysis, using the Log‐Rank model to obtain *p* values. The Cox proportional hazards model was used to assess hazard ratio (HR) values. Overall survival (OS) was defined as the period between diagnosis and the last clinical review or death. *N* = number of patients, HR = hazard ratio.

We studied the association between OS and IL‐11 levels in a retrospective cohort of 106 patients with LUAD from Hospital 12 Octubre (cohort 1, H12O). Clinical outcome information is given in Table S1 in the Supporting Information. Representative images of tumours with positive versus negative IL‐11 protein expression are shown in Figure 1A. Kaplan–Meier curves revealed that IL‐11‐positive staining correlated significantly with poorer OS (*N* = 106, *p* = .001, HR = 2.257 [1.360–3.746]) (Figure 1B). To validate these findings, we performed Kaplan–Meier survival curves using mRNA expression level data from LUAD patients included in TCGA (cohort 2) (Table S2 in the Supporting Information). High IL‐11 expression was clearly related to a poorer OS (*N* = 527, *p* = .040, HR 1.387 [1.014–1.899]) in LUAD patients (Figure 1C), suggesting a role for IL‐11 in cancer progression.^27^

### 3.2 IL‐11 promotes lung adenocarcinoma tumourigenesis

**FIGURE 2:**
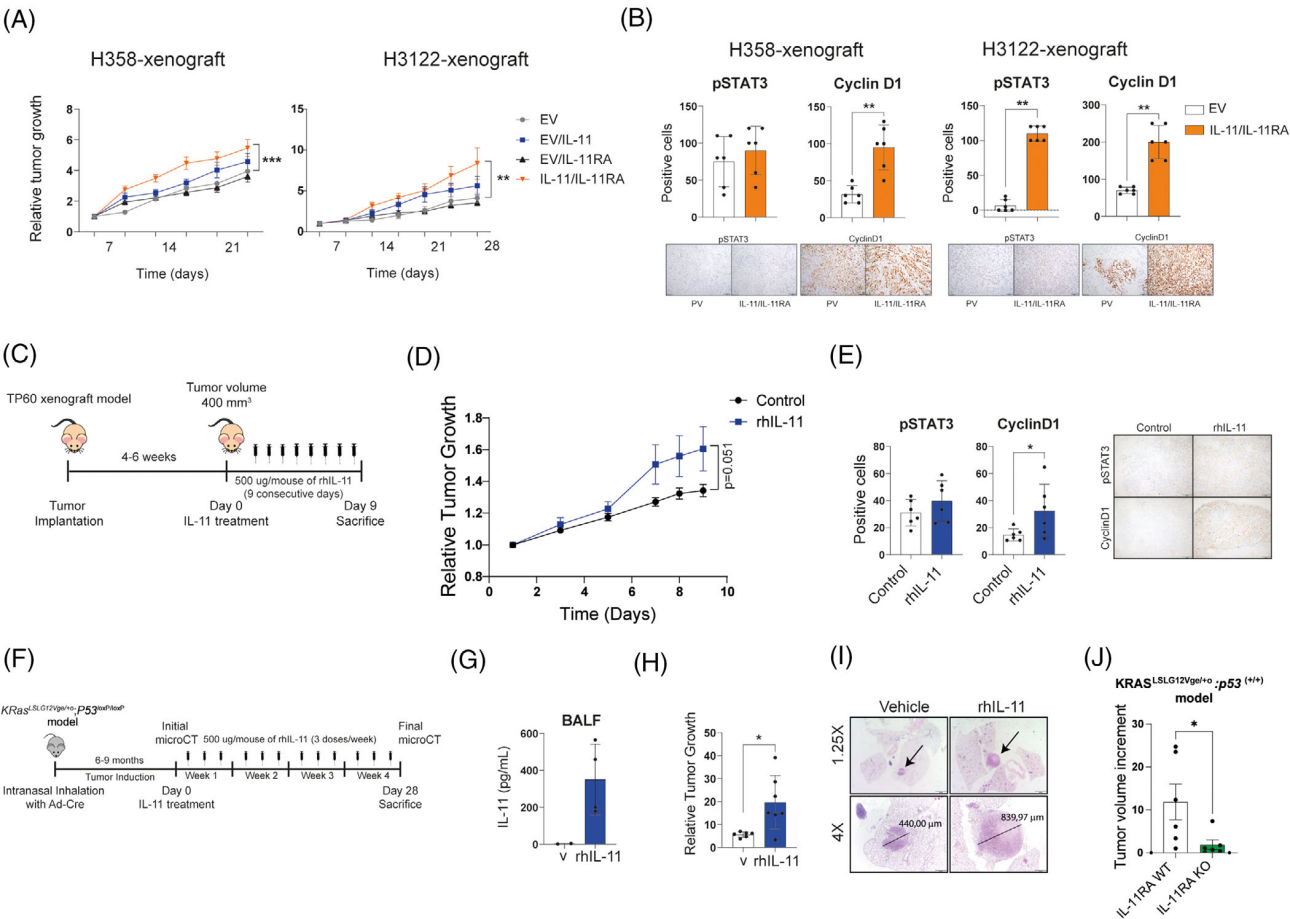
IL‐11 signalling promotes lung cancer tumourigenesis. (A and B) Single cell suspensions of IL‐11‐ or IL‐11RA‐overexpressing lung adenocarcinoma cell lines (H358 and H3122) and a 1:1 mixture of IL‐11 and IL‐11RA variants were subcutaneously engrafted into nude mice. The tumours generated were measured weekly. (A) Xenograft relative tumour volumes of IL‐11‐ or IL‐11RA‐overexpressing cell lines. Five mice per group were included. (B) Quantification of pSTAT3‐ and cyclin D1‐positive cells in the tumour samples were measured by immunohistochemistry. Statistical analysis for (A) was performed by Kruskal–Wallis test; ** (*p* < .01), *** (*p* < .001). EV = cell line with empty vector, IL‐11 = cell line over‐expressing IL‐11, IL‐11RA = cell line over‐expressing IL‐11RA. In (B), *p* values were obtained by Mann–Whitney *U* test ** (*p* < .01). (C) Schematic layout of the experiments in D and E. A LUAD patient‐derived xenografts (PDX, TP60) were engrafted in nude mice. When tumours reached a volume of 400 mm^3^, mice were intraperitoneally injected with rhIL‐11 or vehicle for 9 consecutive days. (D) Relative tumour growth of PDX treated with rhIL‐11 or vehicle (*n* = 5). (E) Quantification of pSTAT3 and cyclin D1 positive cells assessed by IHC from tumour sections of rhIL‐11‐treated mice or control mice. (F) Schematic representation of the experiments in G to I. LUAD were developed in the *KRas^LSLG12Vge/+o^;P53^LoxP/LoxP^* model through intranasal inoculation of adeno‐Cre viruses. Tumour development was monitored with microCT photographing for 6–9 months post virus inoculation. Micro‐CT scans on day 0 were used to stratify mice into treatment groups and to quantify relative response. RhIL‐11 (*n* = 7) or vehicle (*n* = 6) were administered intraperitoneally three times a week for 4 weeks. (G) Bronchoalveolar lavages were collected 3 h after the last rhIL‐11 dosage (*n* = 4) or vehicle (*n* = 2), and hIL‐11 concentration was quantified by ELISA. (H) Size of tumours was measured as the percentage of tumour volume change compared with the baseline tumour volume at day 0. (I) Representative images of H&E staining of lungs from mice treated with vehicle or rhIL‐11 at day 28 post‐virus inoculation. Top panels are 1.25× magnification and bottom panels are 4× magnification. Scale bar 200 µm. Arrows point to tumours. (J) The increase in tumour volume was calculated as the difference between the tumour volume at the end of the experiment and its initial volume. Data and error bars are indicated as mean ± SD. Statistical significance was determined with Mann–Whitney *U* test (*p < .05, **p < .01). H&E = haematoxylin–eosin, V = vehicle, rhIL‐11 = recombinant human IL‐11.

Downstream signalling pathways of the IL‐11/IL‐11RA axis were explored in a panel of nine LUAD cell lines and two non‐tumour cell lines. STAT3 and/or STAT1 activation levels increased in response to IL‐11 in most cell lines, including normal cells. Specifically, this activation is more pronounced in the A549 (STAT1/3), H358 (STAT1), H1650 (STAT3), H1975 (STAT1), Calu‐3 (STAT3), Nuli‐1 (STAT3) and NL20 (STAT1/3) cell lines, while it is weaker in the HCC827, H228, H3122 and H1437 cell lines. (Figure S1A,B in the Supporting Information). H358 and H3122 cell lines were engineered to overexpress either IL‐11 (H358^IL‐11^, H3122^IL‐11^) or IL‐11RA (H358^IL‐11RA^, H3122^IL‐11RA^). Using ELISA, we confirmed the presence of IL‐11 in supernatants of these cell variants (H358^IL‐11^, H3122^IL‐11^) (Figure S2A in the Supporting Information). Increased pSTAT1/3 in response to IL‐11 was observed in cell lines overexpressing IL‐11RA (H358^IL‐11RA^, H3122^IL‐11RA^), as detected by immunoblotting (Figure S2B in the Supporting Information). To evaluate the role of IL‐11/IL‐11RA in tumour growth, single suspensions and 1:1 mixtures of IL‐11‐ and IL‐11RA‐overexpressing cells were subcutaneously engrafted into nude mice. Co‐injection of cells overexpressing IL‐11 and IL‐11RA increased tumour growth significantly with respect to the control group (Figure 2A). Elevated cyclin D expression, downstream target of STAT3, were found in the xenograft models. H3122^IL‐11/IL‐11RA^ xenograft model exhibited a significant increment in pSTAT3 compared with the control group. In the H358IL‐11^/IL‐11RA^ xenograft model, we also found an increment in pSTAT3 levels measured by immunohistochemistry (Figure 2B). These findings suggest that in some context IL‐11 may promote tumour growth via STAT3 activation.

To confirm these results in patient‐derived models, we selected a PDX (TP60) model that expresses low levels of IL‐11 but does express IL‐11RA with minimal basal activation of STAT1 and STAT3 (Figure S3A,B in the Supporting Information). TP60 mice were repeatedly injected with rhIL‐11 for 9 consecutive days (Figure 2C). Exogenous administration of IL‐11 accelerated tumour growth compared with the control group (Figure 2D). Immunohistochemistry staining of tumour sections showed an increase in the levels of pSTAT3 and cyclin D1 in IL‐11‐treated mice (Figure 2E). These data indicate that high levels of IL‐11 contribute to tumour progression.

Next, we assessed whether IL‐11 could exert its pro‐tumourigenic properties in a LUAD genetically modified mouse model. We induced lung adenocarcinomas in a *KRas* ^*LSLG12Vge/+o*^ *;P53* ^*LoxP*/*L* *oxP*^ mouse model by intranasal administration of adeno‐Cre, causing an activation mutation of Kras in parallel with Tp53 inactivation. After the onset of tumours, we administered rhIL‐11 (i.p.) three times a week for 4 weeks (Figure 2F). Detection of hIL‐11 in BALFs in treated mice confirmed its distribution through the respiratory tract (Figure 2G). At the end of the experiment, mice treated with hIL‐11 increased relative average tumour growth compared with control mice (Figure 2H,I). In addition, we demonstrated that *KRas* ^*LSLG12Vge/+o*^;*P53* ^*(+/+)*^ mice developing LUAD tumours but lacking IL‐11RA expression (IL‐11A KO) exhibited significantly smaller tumours compared with those expressing IL‐11RA (IL‐11RA WT), highlighting the role of IL‐11 signalling pathway in tumour growth (Figure 2J). In summary, all these data demonstrated that IL‐11 promotes tumour progression across different LUAD mice models.

### 3.3 Progression of lung adenocarcinoma is attenuated by IL‐11/IL‐11RA knock‐down

**FIGURE 3:**
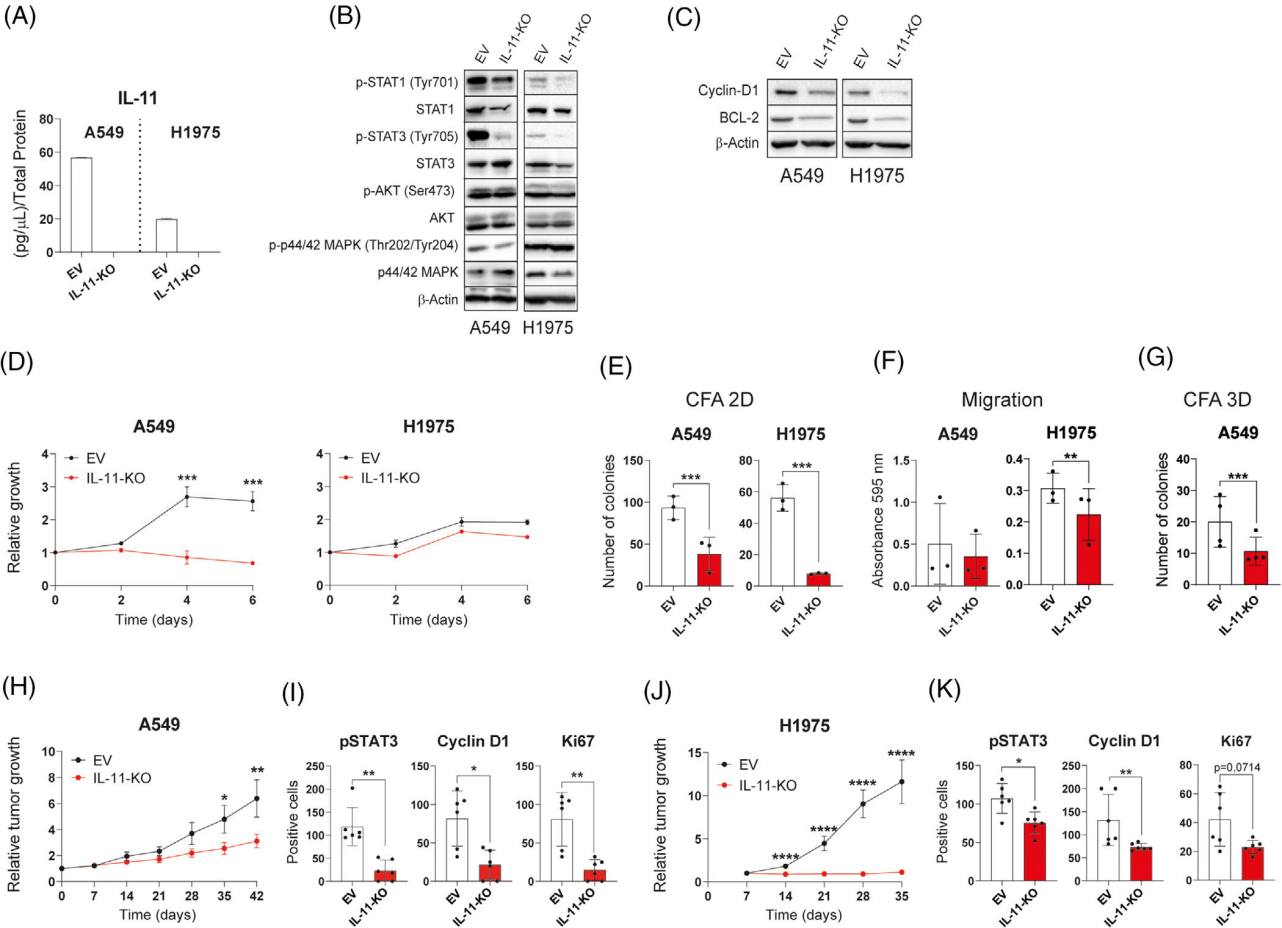
Genetic ablation of IL‐11 reduces pro‐tumourigenic properties in vitro and in vivo. (A) IL‐11 production by IL‐11‐KO and EV genetically modified A549 and H1975 cell lines quantified by ELISA. (B and C) Representative Western blot analysis of pSTAT3 (Tyr705), STAT3, pSTAT1 (Tyr701), STAT1, pMAPK (Thr202/Tyr204), MAPK, pAKT (Ser473), AKT (B) and Cyclin21 and BCL2 (C) on protein extracts of IL‐11‐silenced A549 and H1975 cell lines. (D–G) In vitro surrogate assays were performed to analyse tumourigenic properties: (D) growth curves, (E) colony formation assays (CFA) in 2D, (F) transwell migration assays and (G) 3D soft agar colony formation assay. All experiments were reproduced a minimum of three times in the laboratory and three technical replicates were obtained for each experiment. (H–K) A549^IL‐11KO^ and H1975^IL‐11KO^ and control cell lines were injected subcutaneously into athymic nude mice. (H and J) Relative tumour growth was measured (*n* = 5 mice per group). (I and K) Expression levels of pSTAT3, cyclin D1 and Ki67 were analysed by immunohistochemistry, and we represented the quantification of positive cells of pSTAT3, cyclin D1 and Ki67 tumour sections of xenografts. For growth curves, a representative figure is shown. Means and SDs for the technical replicates are shown on the growth curves. For E to K, data are given as mean + SD. The statistical analysis used was the Mann–Whitney *U* test (*p < .05, **p < .01, ***p < .001). EV = cell line with empty vector, IL‐11‐KO = cell line with IL‐11 silencing.

To understand whether lung cancer cells depend on IL‐11 for tumour progression, we selected two LUAD cell lines (A549, H1975) that express high levels of IL‐11 by ELISA (Figure S1C in the Supporting Information) and disrupted the IL‐11 gene by CRISPR/Cas9 technology (Figure 3A). The absence of IL‐11 led to reduced phosphorylated STAT1 and STAT3 (Figure 3B), decreased the proliferation marker Cyclin D1, the anti‐apoptotic protein Bcl‐2 (Figure 3C). Genetic deletion of IL‐11 significantly reduced growth of the A549 cell line but not that of the H1975 cell line (Figure 3D). IL‐11‐knockout in both cell lines resulted in a reduced clonogenicity in 2‐ and 3D (when cells can grow in this format), and a decreased migration ability compared with controls (Figure 3E–G).

Xenograft models were established by subcutaneous injecting IL‐11 CRISPR/Cas9 silenced cell variants or the parental cell line transfected with an EV. The absence of IL‐11 significantly reduced the relative tumour growth in both mouse models (Figure 3H,J). Slower tumour growth rate was accompanied by a reduced expression of Ki67 and cyclin D1 and a lower accumulation of pSTAT3 (Figure 3I–K). Taken together, these results corroborate the role of IL‐11 in the progression of LUAD.

To further validate the requirement for IL‐11 signalling in LUAD progression, we used CRISPR/Cas9 technology to silence IL‐11RA in the same cell lines (Figure S4A in the Supporting Information). IL‐11RA silencing resulted in reduced phosphorylated STAT1 and STAT3 status (Figure S4B in the Supporting Information) and a slower cell growth rate (Figure S4C in the Supporting Information). Migration and soft agar assays indicated a decreased migration and invasive capacity of IL‐11RA knockdown cell variants compared with controls (Figure S4D–F in the Supporting Information).

For in vivo assessment of cancer cells growth linked to IL‐11 activity, we established subcutaneous xenografts of A549 and H1975 cells knocked down for IL‐11RA (Figure S4G–I in the Supporting Information). IL‐11RA knockdown A549 tumour‐bearing mice exhibited a significant lower tumour burden (Figure S4G in the Supporting Information). Genetic abrogation of IL‐11RA signalling in the A549 xenograft was accompanied by a reduction in STAT3 activation along with a limited cell proliferation, as measured by cyclin D1 and Ki67 expression (Figure S4H in the Supporting Information). However, these effects were not observed in the H1975 xenografts (Figure S4I in the Supporting Information).

These results suggest that IL‐11RA inhibition in tumour cells could be a potential therapeutic strategy.

### 3.4 Therapeutic targeting of IL‐11/IL‐11RA reduces tumour growth

**FIGURE 4:**
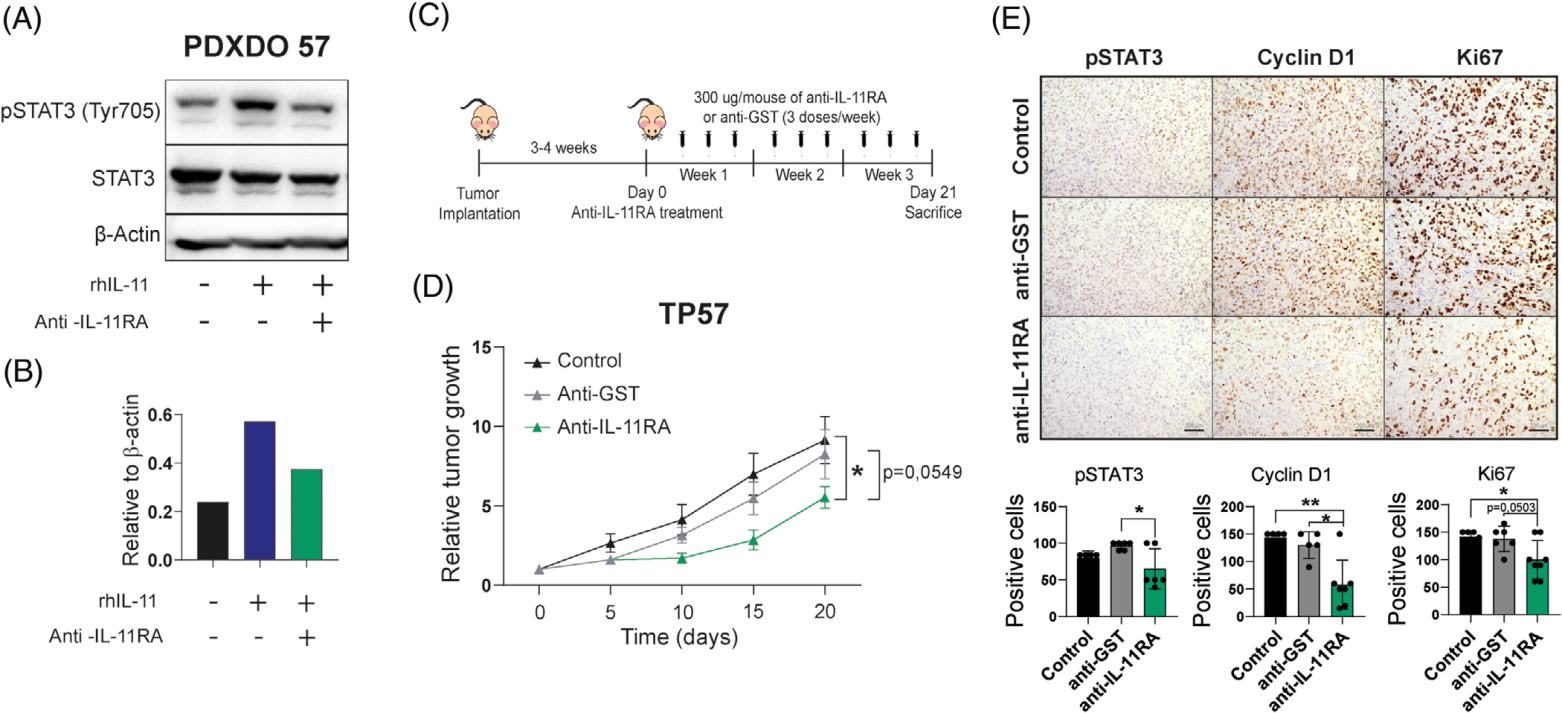
Therapeutic inhibition of IL‐11/IL‐11RA signalling with a neutralising anti‐IL‐11RA antibody reduces tumour growth in a PDX model. (A) Three‐dimensional organoids were generated from a lung adenocarcinoma PDX model (PDXDO from TP57) and were treated with rhIL‐11 and anti‐IL‐11RA Ab (1000 µg/mL). Representative Western blot analysis of pSTAT3 (Tyr705) on protein extracts from PDXDO of TP57 pre‐incubated with the neutralising antibody against hIL‐11RA for 24 h before stimulation with rhIL‐11. (B) Densitometry quantification of the reactive bands for pSTAT3 (Tyr705) and STAT3 and relative expression were normalised to the total amount of β‐actin. Data are shown as mean. (C) Schematic layout of the experiments in D to E. PDX tumours were engrafted in both flanks of athymic mice (PDX 57). When a volume of 200 mm^3^ was reached, mice were treated with anti‐IL‐11RA mAb (300 µg/mice), anti‐GST mAb (300 µg/mice) or vehicle by intraperitoneal injection, three times a week for 3 weeks (*n* = 5). (D) Relative tumour growth of PDX TP57 normalised to the tumour volume at baseline (day 0). (*E*) Positive cells expressing pSTAT3, Ki67 and Cyclin D1 in tumour sections were analysed by immunohistochemistry and quantified. Representative microphotographs and quantifications are shown. Scale bar 200 µm. Data are given as mean + SD. Statistical significance was determined with the Kruskal–Wallis test (*p < .05, **p < .01, ***p < .001).

We generated an IL‐11RA neutralising antibody (mAb) (Figure S5A in the Supporting Information) that efficiently blocks the IL‐11/IL‐11RA signalling pathway in the A549 cell line and the organoids derived from de PDX TP57 as shown the reduction of p‐STAT1 and p‐STAT3 by WB (Figures S5C in the Supporting Information and 4A,B). Treating a high IL‐11 PDX model (TP57) (Figure S3A in the Supporting Information) with the IL‐11RA mAb, following the scheme shown in Figure 4C, decreased tumour size compared with anti‐GST (glutathione‐S‐transferase) mAb‐treated or control animals (Figure 4D). The IL‐11/IL‐11RA signalling pathway and tumour proliferation were significantly down‐regulated as assessed by IHC staining of pSTAT3, and cyclin D1 and Ki67, respectively (Figure 4E). These data demonstrated that anti‐IL‐11RA mAb blocks LUAD progression, acting as a potential anti‐tumour therapy in patient‐derived mouse models.

### 3.5 Impact of IL‐11 on the TME in tobacco‐exposed lung cancer mouse models

**FIGURE 5:**
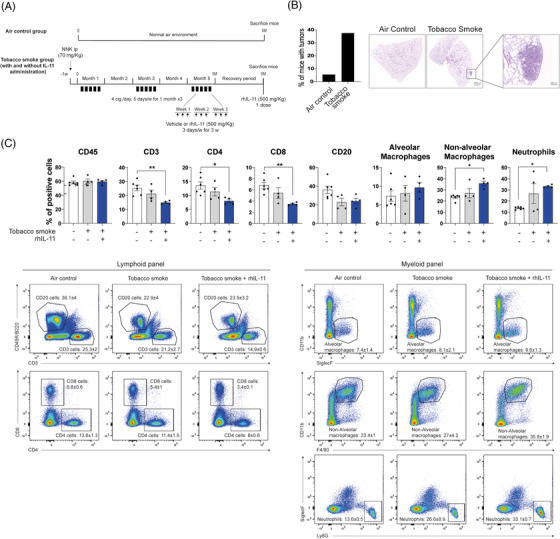
Effect of IL‐11 in the tumour microenvironment in a tobacco‐exposure lung cancer mouse model. (*A*) Schematic layout of the experiments in b to c. The air control group was exposed to a normal air environment. Tobacco smoke group belongs to A/J mice intraperitoneally injected with NNK (4‐(Methylnitrosamino)‐1‐(3‐Pyridyl)‐1‐Butanone) 1 week before initiation of MTS (mainstream tobacco smoke) exposure. On the fifth month after exposure, half of mice were injected with rhIL‐11, three times per week for 3 weeks. Tumour development was analysed 9 months after tobacco‐exposure initiation. (*B*) Percentage of mice per group with lung tumours. Histology (H&E stain) in A/J mice 9 months after initiation of the tobacco‐exposure protocol. Scale bar 1000 and 200 µM. (C) Lungs from IL‐11 treated mice were analysed by flow cytometry for the frequencies of infiltrating lymphocytes: CD3 T cells (Live/Dead^−^ CD45^+^ CD3^+^); CD4 T cells (Live/Dead^−^ CD45^+^ CD3^+^ CD4^+^); CD8 T cells (Live/Dead^−^ CD45^+^ CD3^+^ CD8^+^); B cells (Live/Dead^−^ CD45^+^ CD3^−^ CD20^+^); alveolar macrophages (Live/Dead^−^ CD45^+^ B220^−^ Ly6G^−^ SiglecF^+^); non‐alveolar macrophages (Live/Dead^−^ CD45^+^ B220^−^ Ly6G^−^ CD11b^high^ F4/80^+^) and neutrophils (Live/Dead^−^ CD45^+^ B220^−^ Ly6G^+^). Data are given as mean + SD. Representative dot plots are shown for groups. Statistical significance was determined with the Kruskal–Wallis test ** (*p* < .01), *** (*p* < .001).

We examined IL‐11/IL‐11RA axis mediated changes in the lung TME using tobacco‐induced LUAD murine models (Figures 5A and S7A in the Supporting Information), which are immunocompetent models that better approximate the clinical reality of smoking‐related lung cancer. As expected, the percentage of A/J mice that developed lung tumours was higher in TS‐exposed (40%) mice compared with those exposed to air (10%) (Figure 5B). We confirmed the accumulation of hIL‐11 in BALFs from mice exposed to TS and treated with IL‐11 (i.p) compared with mice exposed to TS alone or the control group (Figure S6A in the Supporting Information). Interestingly, the anti‐tumoural cytokine IL‐12p70 decreased to undetectable values in BALFs from tobacco‐smoke rhIL‐11‐treated mice (Figure S6B in the Supporting Information). Flow cytometry revealed a significant loss of T cell populations (CD3, CD4 and CD8) in mice exposed to TS and treated with IL‐11 compared with control mice. We also observed a trend towards B cell exclusion from the lung parenchyma in response to TS exposure and independent of IL‐11. Alveolar macrophages were not significantly affected, but non‐alveolar macrophages and neutrophils were up‐regulated rhIL‐11 and TS‐exposed mice (Figure 5C). Same results were observed in a similar experiment carried out in the genetic mouse model *KRas^LSLG12Vge/+o^;P53^(+/+)^* (Figure S7A,B in the Supporting Information). These results indicate that IL‐11 contributes to the immune evasion of T cell populations and favours macrophage recruitment into the lungs.

### 3.6 IL‐11 leads to an altered TME in LUAD patient samples

**FIGURE 6:**
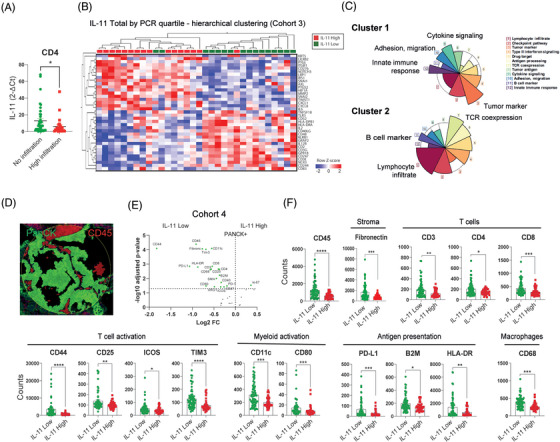
IL‐11 abundance is associated with a skewed tumour microenvironment in LUAD patients’ samples. (A) IL‐11 expression determined by RT‐qPCR was compared between the patient groups in cohort 3: those with null CD4 infiltration *N* = 39 and those with high CD4 infiltration *N* = 25, as assessed by immunohistochemistry. (B) Heatmap of changes in expression of a group of genes analysed by targeted RNASeq (OIRRA panel). The expression profile from this set of 37 genes, segregates the samples according to a high (red) or low (green) expression of IL‐11 in patients with lung adenocarcinoma (cohort 3; HU12O, *n* = 67). FDR ≤ .1. (C) Over‐representation analysis (ORA) associating onco‐immune functions. Each coloured sector represents a functional category whose size indicates over‐ or‐under representation of genes in that cluster within a specific function. (D) Example of a selected region of interest (ROI) (Å∼200 µm) from a representative patient with advanced lung adenocarcinoma. A mask for immune cells was generated based on CD45 staining (red) and another mask for tumour was created based on PanCytokeratine staining (green) using GeoMx DSP. (E) Volcano plot of differentially expressed markers obtained by unpaired test with a Benjamini–Hochberg correction from the tumour compartment (PanCK+) of high‐IL‐11 versus low‐IL‐11 LUAD patients (cohort 4; HU12O, *n* = 20). Tumour samples were separated based on IL‐11 expression detected in tumour cells by immunohistochemistry and classified as low‐IL‐11 [IL‐11≤10% (*N* = 12)] or high‐IL‐11 [(IL‐11 > 10% (*N* = 8)]. (F) Total counts of immune cell markers of high‐IL‐11 and low‐IL‐11 expressing tumour samples patients. *p* Values were obtained by Mann–Whitney *U* test (*p < .05, **p < .01, ***p < .001).

To further evaluate the role of IL‐11 in the TME, we assess the possible correlation between the presence of various immune cell populations and IL‐11 expression measured by RT‐qPCR from frozen patient samples from cohort 3 (M&M section). We divided the patients into two groups based on their low/null or high immune cell infiltration (CD4, CD8, CD20 and CD68) and analysed the IL‐11 expression levels in both groups. We found that patients expressing high levels of IL‐11 showed statistically significant lower CD4 infiltration (Figure 6A) but not with the rest of the immune populations (Figure S9 in the Supporting Information).

In the same cohort, we also analysed the expression of 395 genes reported to be involved in tumour‐immune interactions using a targeted RNA‐Seq panel (OIRRA). The gene expression data of the 16 IL‐11‐high expressing samples and the 16 IL‐11‐low expressing samples were evaluated using Qlucore Software and unsupervised hierarchical clustering was performed. We found 37 differentially expressed (FDR < 0.1) genes that defined two subgroups of LUAD tumours with distinct transcriptomic profiles (Figure 6B). Subgroup one was enriched with tumour samples expressing high levels of IL‐11 (>75%, *n* = 16) whereas subgroup two was enriched in tumour samples with low levels of IL‐11 expression (<25%, *n* = 16). Most up‐regulated genes in subgroup one was implicated in functions related to tumourigenesis such as epithelium–mesenchymal transition (EMT), migration, cell invasion and survival of cancer cells (ITGB1, SNAI1, AXL, HIF1A, SNAI2, LILRB2, TWIST1), and cytokines and chemokines involved in the recruitment of myeloid cells and neutrophils (CXCL8, CXCL1, IL‐6 and CD276). Subgroup two up‐regulated genes related to the development and activation of T cells (CD3E, CD3G, TNFRSF18, CD247, CD244), cytotoxic activation of NK cells (NCR3), immune activation cytokines (IL‐2, IL‐12), and dendritic cell activation (CD83, HLA‐DPB1, HLA‐DRA, CD40L). Over‐representation analysis (ORA) confirmed that the gene cluster 1 is up‐regulated in IL‐11 High subgroup and is over‐represented by oncogenic functions, while gene cluster 2 is up‐regulated in IL‐11 Low subgroup and is over‐represented by immunogenic genes (Figure 6C).

Further, we examined the expression of immune proteins in the tumour compartment (PanCK+) using DSP technology (Figure 6D). A volcano plot showed significantly elevated expressions of immune markers (*p* adjusted < .05 with BH correction) in the tumour compartment from samples with low IL‐11 expression levels (IL‐11 Low) compared with those with high IL‐11 (IL‐11 High) expression levels (Figure 6E). Low expression of IL‐11 in tumour cells was associated with significantly higher levels of leukocytes (CD45), T cells (CD3, CD4, CD8), T‐cell activation (CD44, CD25, ICOS, Tim3), dendritic cell activation, antigen presentation (CD11c, CD80, B2M, HLA‐DR) and macrophage (CD68) markers (Figure 6F). Of note, Ki67 was the only marker found to be up‐regulated in patients showing high levels of IL‐11 expression in the volcano plot. Overall, the immune profiling analysis indicates that high levels of IL‐11 may modulate the TME, skewing the immune cell infiltration, promoting immune evasion and impairing the anti‐tumour response.

## 4 DISCUSSION

Emerging evidence suggests that IL‐11 plays a significant role in various lung diseases, with its involvement recently demonstrated in idiopathic pulmonary fibrosis,^28^ and its impact on other lung pathologies gradually being uncovered. Our findings indicate that IL‐11 has a pro‐oncogenic effect in LUAD and is linked to poorer prognosis. Persistent activation of IL‐11/IL‐11RA signalling drives a dysregulated TME leading to immune evasion. We propose that the therapeutic inhibition of IL‐11 might be effective in reducing tumour burden in LUAD patients expressing high levels of IL‐11.

Previous work from our group showed that BALFs from LUAD patients accumulated significant levels of IL‐11 compared with non‐LUAD patients, suggesting a potential role for IL‐11 as a diagnostic marker.^15^ To study the role of IL‐11 as a prognostic biomarker, we examined two cohorts of patients with early‐stage lung adenocarcinoma. Our data showed that an elevated expression of IL‐11 correlated with a worse prognosis in both patient cohorts. These results are in line with another study in which IL‐11 expression levels from lung adenocarcinoma patients correlated with a poorer prognosis,^20^ indicating that IL‐11 may play a role in tumour progression.

Published data have shown a clear link between the IL‐11/IL‐11RA axis and a hyperactivation of STAT3 signalling leading to anti‐apoptotic, proangiogenic, migration and aggressiveness for gastric,^29^, ^30^ colorectal,^31^, ^32^ endometrium,^33^ breast,^17^ glioblastoma^34^ and lung cancer.^20^ Here, we present evidence that IL‐11 plays a major role in exacerbating tumour cell growth mediated through STAT3 and STAT1 activation. We observed that genetic ablation of IL‐11 or IL‐11RA produced a clear reduction of the STAT1 and STAT3 activation signalling pathways. Consequently, inhibition of the JAK/STAT pathway by IL‐11/IL‐11RA disruption resulted in a significant reduction of oncogenic properties, reduced tumour growth of lung adenocarcinoma xenografts and decreased susceptibility to lung cancer in a GEMM model with IL‐11RA knockout in both tumour and normal cells. We observed that tumour cells activate the STAT3 and STAT1 signalling pathways, but not the PI3K–AKT or MAPK pathways, after the IL‐11 challenge. In addition, we detected a decrease in cyclin D1 and BCL2 proteins, which have been described as targets of STAT1^35^ and STAT3.^20^, ^36^ However, cyclin D1 and BLC2 are also regulated by the AKT pathway. While the PI3K–AKT–mTORC1 and MAPK pathways promote tumourigenesis by enhancing proliferation, suppressing apoptosis and facilitating metastasis, the role of the IL‐11–PI3K–AKT axis in cancer development remains poorly understood. Despite this, the IL‐11/IL‐11RA axis triggers fibroblast activation and extra‐cellular matrix (ECM) production via the ERK signalling pathway. Thus, we hypothesise that IL‐11 mainly exerts its intrinsic pro‐tumourigenic role via STAT3 activation, whereas the PI3K–AKT–mTORC1 or MAPK pathways may be triggered by IL‐11 from other cells in the tumour stroma, thus favouring tumour progression. Notably, these results converge with those of recent work indicating a role for the CLCF1–CNTFR signalling axis in lung cancer.^37^

A malignant tumour is more than an aggregate of tumour cells; it forms a complex microenvironment comprising fibroblasts, infiltrating immune cells, endothelial cells and structural components that impact tumour development, metastasis and progression.^38^ However, the role of IL‐11 in the TME from LUAD remained unknown. Our data show that the IL‐11/IL‐11RA axis from tumour cells conducts the TME towards an immunosuppressive landscape and indicate that IL‐11 modulates the TME mainly by impairing T cell infiltration and by incrementing macrophage levels. Previous data from our group showed that IL‐11 was highly expressed in BALFs from LUAD patients.^15^ We therefore determined the levels of different cytokines in mice exposed to TS. Interestingly, we found that the expression of IL‐12p70 was significantly lower in BALF from mice exposed to TS plus IL‐11, suggesting a possible oncogenic and immunosuppressive mechanism by IL‐11‐stimulated alveolar macrophages.^39^, ^40^ However, it is not known whether IL‐11‐primed TAMs create a tumourigenic niche.

Additionally, we confirmed and expanded these immune‐modulating findings in tumour samples from LUAD patients. Two gene expression patterns according to IL‐11 expression levels were obtained. In the subgroup of patient tumour samples with an elevated accumulation of IL‐11, EMT genes (SNAI1, AXL and TWIST1) and hypoxia factor 1 (HIF1A) were over‐expressed compared with the subtype with a lower accumulation of IL‐11. It has previously been reported that IL‐11 promotes tumour progression and EMT in lung adenocarcinoma through the activation of the STAT3/HIF‐1α/EMT signalling pathways.^41^ In line with this, other studies demonstrated a critical role of STAT3 activation through IL‐6/IL‐11 in cancer‐associated fibroblasts (CAFs) in the promotion of colorectal cancer development^42^, ^43^

Consistent with these results, our DSP analysis reinforced the idea that in the absence of IL‐11 the immune response is boosted, as shown by the higher level of markers related to T cells, T‐cell activation and myeloid cell activation and maturation. One of the mechanisms proposed for IL‐11 mediating immune evasion is a suppression of the anti‐tumoural functions of CD4^+^ Th1 cells, thereby favouring tumour progression in a mouse model for colon adenocarcinoma.^44^ Nevertheless, we cannot exclude additional immunomodulatory effects of IL‐11 given that most immune cells (dendritic cells, macrophages, NKs and B cells) express IL‐11RA. CAFs are a major component of the TME and one of the main sources of IL‐11^43^. IL‐11 could function dually, autocrine and paracrine promoting profibrotic fibroblast effector activity and myofibroblast differentiation via an ERK‐dependent mechanism. Additionally, fibroblasts stimulated by IL‐11 develop resistance to cell death by secreting proinflammatory chemokines and cytokines (such as IL‐6, CCL2, CXCL1). This effect leads to the recruitment and activation of immune cells to the tumour site.^45^, ^46^ Therefore, high levels of IL‐11 are associated with a complex microenvironment that drives tumour progression and immunosuppression. However, the mechanism by which IL‐11 mediates immunosuppression in tumours remains unknown.

Previous studies have demonstrated that the pharmacological blockade of IL‐11 signalling has anti‐tumour effects.^47^ In a mouse model for colitis‐associated cancer^48^ and gastric carcinomas.^49^ IL‐11 mutein binds to the IL‐11R/gp130 complex and blocks IL‐11/IL‐11RA signal transduction, reducing the tumour growth. Other authors have shown similar anti‐tumour effects with an IL‐11RA neutralising antibody in endometrial tumours.^50^ Another study also demonstrated that BMTP‐11, a drug targeting IL‐11RA, reduced osteosarcoma tumour growth and lung metastasis formation, although this drug was associated with renal toxicity.^51^ In line with this, one report on osteosarcoma lung metastases showed that CAR‐T cells against IL‐11RA decreased tumour growth in xenograft models.^52^ However, none of the mentioned studies used PDX models, which would likely be more appropriate by preserving the human tumour properties. Our findings suggest that neutralising IL‐11RA mAb opens a new therapeutic strategy for lung adenocarcinoma patients and possibly for other types of inflammation‐related cancers. In this regard, our work provides further knowledge and therapeutic tools to treat patients expressing high levels of IL‐11. Some studies have highlighted that targeting a single member of the IL‐6 family might not be sufficient.^53^ This leads to the possibility of combinatorial blockade strategies targeting two members of the IL‐6 family in order to improve efficacy.^54^ Our OIRRA data showed that tumour samples that express high levels of IL‐11 also up‐regulate IL‐6 expression, potentially triggered by TGFB. However, the direct targeting of TGFB has major side effects and no effective treatment has been developed. Based on this, we hypothesise that blocking IL‐11 and IL‐6 may have synergic outcomes and improve the anti‐tumour benefit of drugs. As our DSP analysis also demonstrated, tumour samples from LUAD patients that express low levels of IL‐11 showed increased levels of immune expression markers, implying that such patients could be more susceptible to receive immunotherapy treatment. Nevertheless, this therapy does not appear to be appropriate for LUAD patients expressing high levels of IL‐11. In order to identify appropriate strategies to treat such patients, further studies are needed to decipher the mechanism of IL‐11 in tumour cells and the stroma compartment, and of the nature of IL‐11's crosstalk with the TME. Applying this concept in clinical setting may lead to the development of more rational patient‐tailored novel therapies to be developed that target IL‐11 and improve clinical outcomes for patients.

## AUTHOR CONTRIBUTIONS

*Conceptualisation*: I.F., A.C. and L.P.‐A. *Methodology*: L.O., C.C., P.Y., A.S., J.R., R.S., P.C., P.P., A.R., M.T.M., J.L.S., G.R., P.G., R.G.‐L., E.A.S.C., M.B. and R.M. *Software*: C.C., E.M.G.‐M., J.R., A.R. and J.Z. *Formal analysis*: I.F, L.O., C.C., E.M.G.‐M., A.S. and A.R. *Validation*: C.C, A.S., P.C. *Visualisation*: L.O., C.C., E.M.G.‐M., I.O. and I.F. *Investigation*: L.O., C.C., I.O., S.M.‐P. and I.F. *Resources*: I.F. and L.P.‐A. *Data curation*: E.M.G.‐M. and J.R. *Writing – original draft preparation*: L.‐O., C.C., G.R. and I.O. *Writing – review and editing*: C.C., I.O., S.M.‐P., A.C., I.F., E.A.S.C. and L.P.‐A. *Supervision*: I.F. and L.P.‐A. *Project administration*: I.F. and L.P.‐A. *Funding acquisition*: I.F. and L.P.‐A. All authors have read and agreed to the published version of the manuscript.

## CONFLICTS OF INTEREST STATEMENT

L.P.‐A. has received honoraria for scientific advice and speaker fees from Lilly, Merck Sharp & Dohme, Bristol‐Myers Squibb, Roche, PharmaMar, Merck, AstraZeneca, Novartis, Boehringer Ingelheim, Celgene, Servier, Sysmex, Amgen, Incyte, Pfizer, Ipsen, Adacap, Sanofi, Bayer and Blueprint, and participates as an external member on the board of Genómica. He is founder and board member of Altum sequencing and has received institutional support for contracted research from Merck Sharp & Dohme, Bristol‐Myers Squibb, AstraZeneca and Pfizer. J.Z. reports personal fees from Sanofi, Pfizer, Novartis, Guardant Health, Takeda and NanoString and grants and personal fees from AstraZeneca, Roche and Bristol Myers Squibb outside the submitted work. EASC reports grants from NCI and St Baldricks Foundation; as well as grants from Hyundai Hope on Wheels during the conduct of the study. The remaining authors declare no potential conflicts of interest.

## Supporting information



Supporting Information

Supporting Information

Supporting Information

Supporting Information
