# Supplementary material for: Interleukin‐11 promotes lung adenocarcinoma tumourigenesis and immune evasion
Source: Clin Transl Med. 2025 Jul 17;15(7):e70374. doi: 10.1002/ctm2.70374 (PMC12268789; doi:10.1002/ctm2.70374)
Supplement: Supplementary file 1 — Supporting Information [file CTM2-15-e70374-s001.docx]

**Supporting Information**

**Title**

**IL-11 promotes lung adenocarcinoma tumorigenesis and immune evasion.**

**Authors**

**Cristina Cirauqui^1,2,3^ *, Laura Ojeda^1,2,^ *, Itziar Otano^1,2^, Irene Pazos^1,3^, Alba Santos^1,2^, Eva M Garrido-Martín^1^, Patricia Yagüe^1,2^, Javier Ramos-Paradas^1,2^, Sonia Molina-Pinelo^2,4^, Giovanna Roncador^5^, José Luis Solórzano^1,6^, M Teresa Muñoz^1^, Patricia Cozar^1^, Patricia Plaza^1^, Rocío Suárez^1,2^, Marta Jiménez^1^, Roberto Moreno^1^, Arantxa Rosado^1^, Pablo Gámez^7^, Ricardo García-Luján^7^, Jon Zugazagoitia^1,2,8^, E. Alejandro Sweet-Cordero^9^, Mariano Barbacid^10^, Amancio Carnero^2,4^, Irene Ferrer^1,2,3^ * ^#^, Luis Paz-Ares^1,2,8,11,^***

**Supplementary Figures and tables**


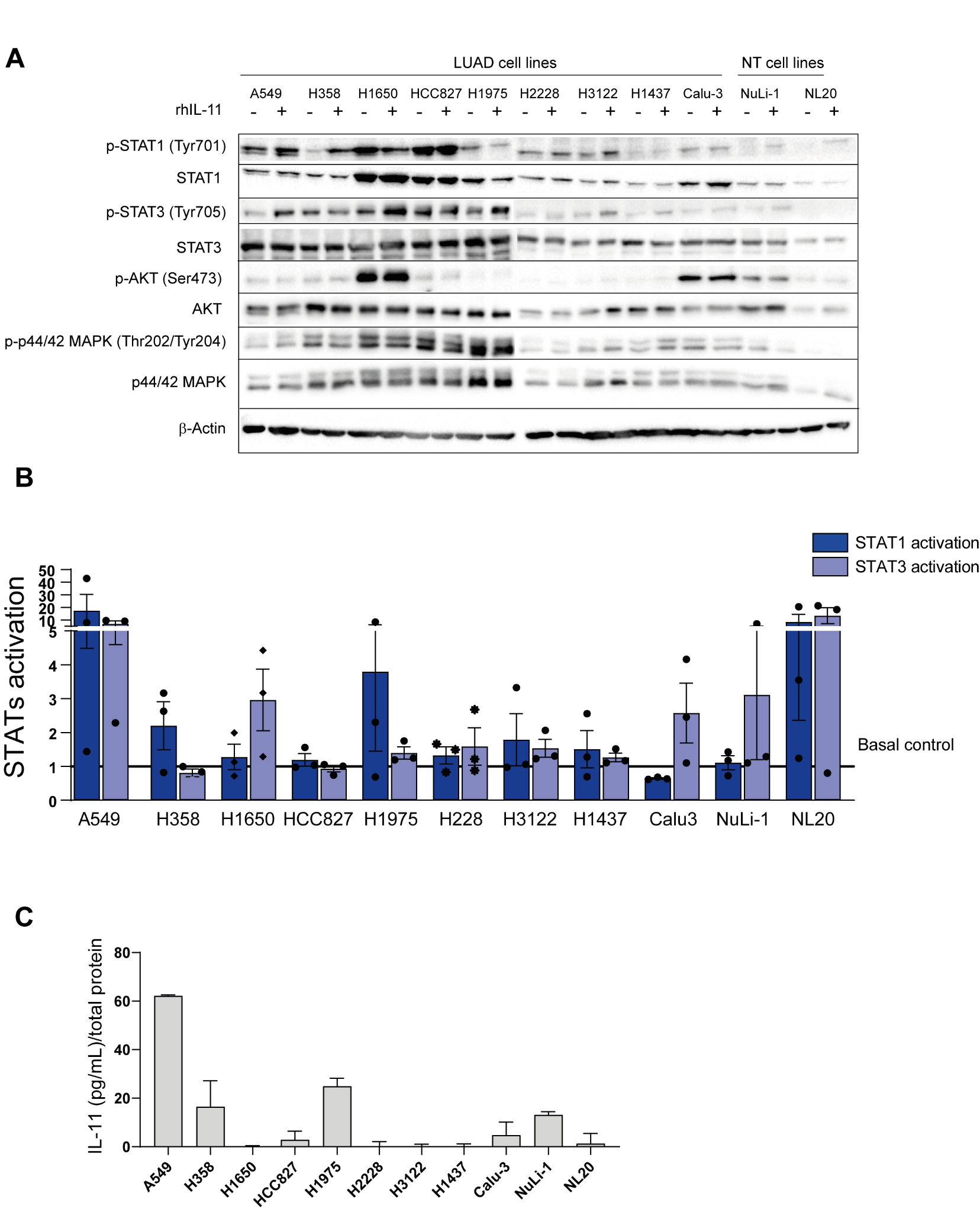


**Figure E1**

**Figure E1 Effect of IL-11 in the LUAD cells lines.** (*A*) IL-11 stimulation triggers STAT3 and STAT1 activation in lung adenocarcinoma. Cell lines were stimulated with 50 ng / ml of rhIL-11, for 15 minutes (+). After stimulation, activation of STAT1, STAT3, p44/42 MAPK and AKT were analyzed by western blot in the protein extracts. (*B*) Quantification of STAT1 and STAT3 activation from all three western blot replicates. The normalization of band quantification was performed for each phosphorylated protein relative to the total one and, in turn, to each cell line without stimulation with rhIL-11. Values greater than 1 correspond to increases in the activation of the analyzed protein in the rhIL-11-stimulated cell line compared to the same unstimulated cell line. (*C*) Concentrations of human IL-11 measured by ELISA in the panel of the cell lines. NT = non-tumoral. (*C*) rhIL-11 = recombinant human IL-11 protein.


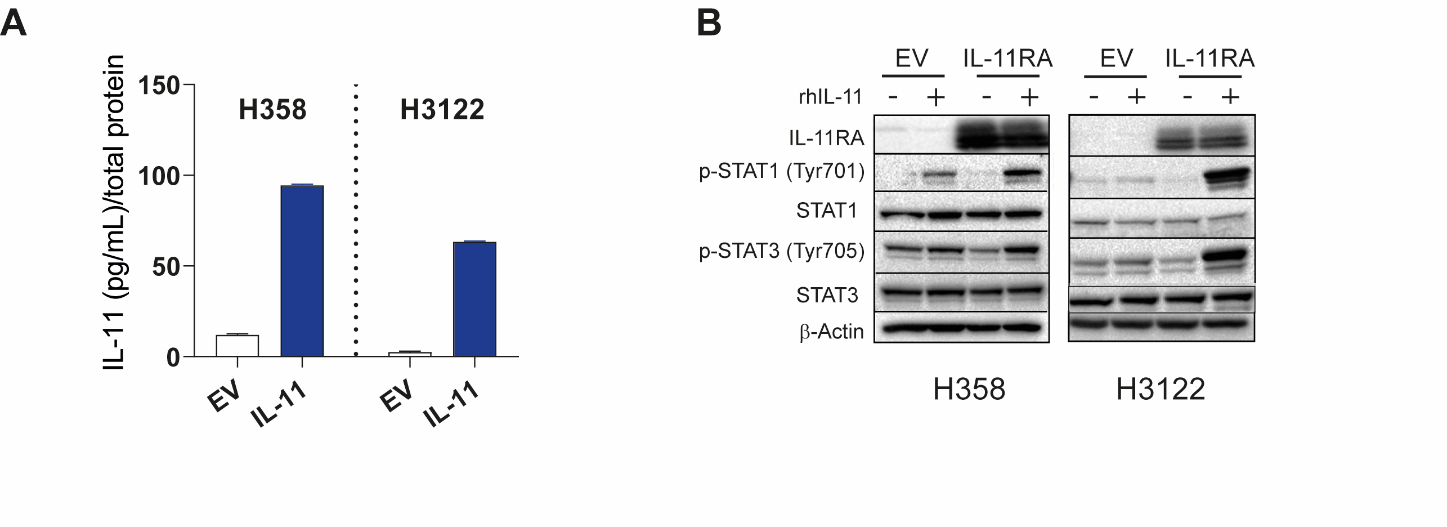


**Figure E2**

**Figure E2 Generation of IL-11 or IL-11RA overexpressing cell lines.** (*A*) ELISA quantification of IL‐11 in supernatants produced by IL-11-overexpressing H358 and H3122 cells compared to empty-vector-expressing cells. (*B*) Representative Western Blot analysis of pSTAT3 (Tyr705), STAT3, pSTAT1 (Tyr701) and STAT1 extracts from H358^IL-11RA^ and H3122 ^IL-11RA^ cell lines. EV = empty plasmid cell line, IL-11 = IL-11 over-expressing cell line, IL-11RA = IL-11RA over-expressing cell line, rhIL-11 = recombinant human IL-11 protein.


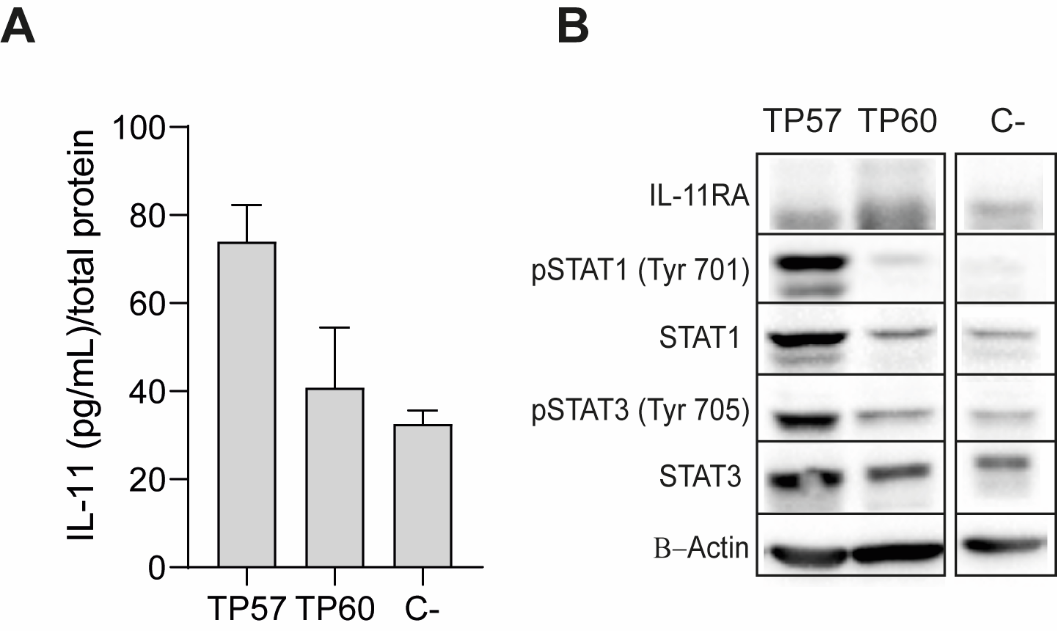


**Figure E3**

**Figure E3 Molecular characteristics of the two PDX models employed.** (*A*) Concentrations of human IL-11 measured by ELISA from tumors derived of PDX57 and PDX60 compared to a negative control PDX for IL-11 expression. (*B*) Western Blot analysis of pSTAT3 (Tyr705), STAT3, pSTAT1 (Tyr701) and STAT1 tumors extracts from PDX57 and PDX60 compared to a negative control PDX, the model from our PDX collection with the least activated STATs pathways.

**
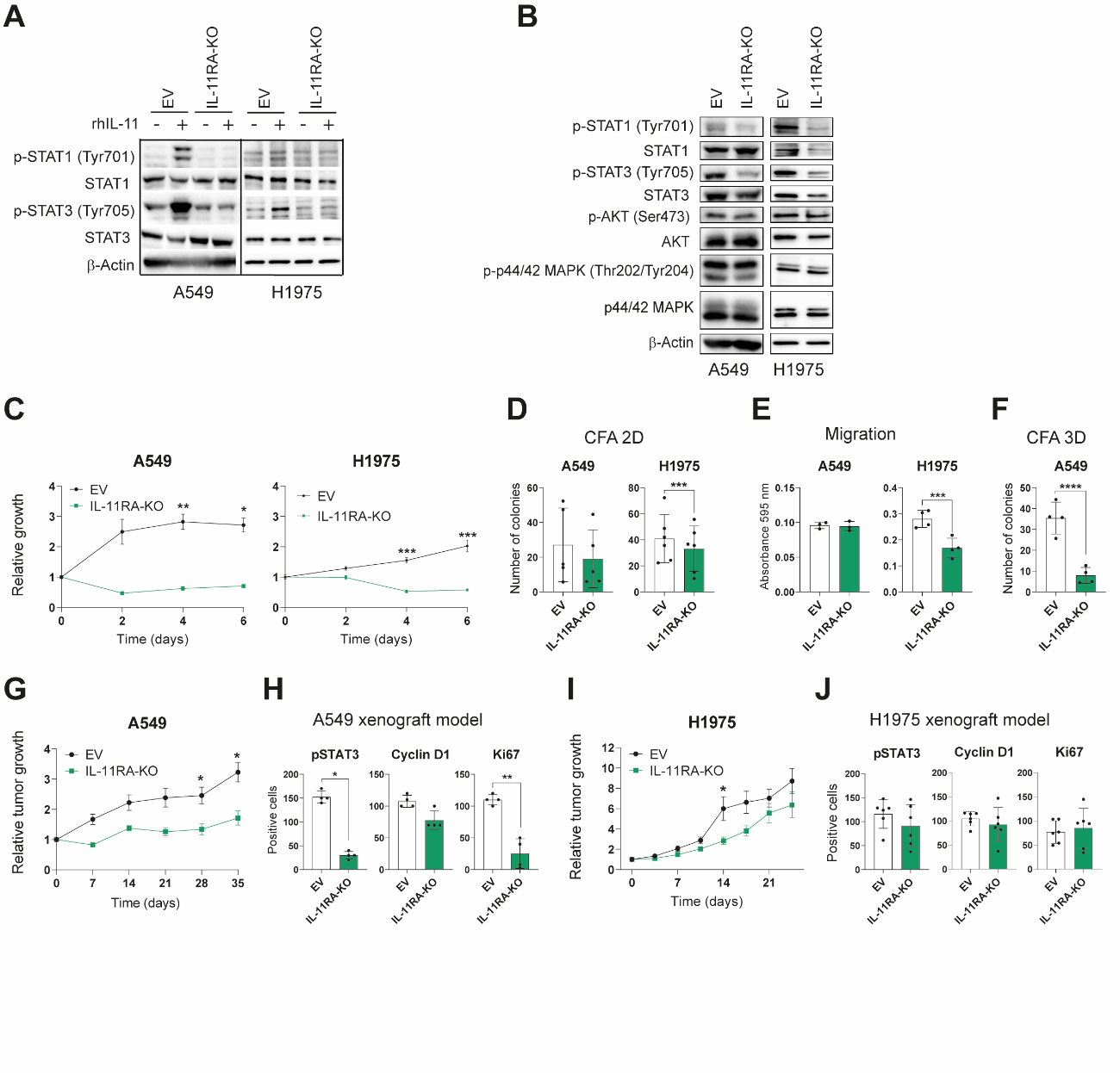
**

**Figure E4**

**Figure E4 Genetic ablation of IL-11RA reduces tumor cell proliferation *in vitro* and *in vivo*.**

(*A*) Representative Western Blot analysis of pSTAT3 (Tyr705), STAT3, pSTAT1 (Tyr701) and STAT1, on protein extracts of IL-11RA-silenced A549 and H1975 cell lines after stimulation with rhIL-11 (50 ng/ml) for 15min. (*B*) Representative Western Blot analysis of pSTAT3 (Tyr705), STAT3, pSTAT1 (Tyr701), STAT1, pMAPK (Thr202/Tyr204), MAPK, pAKT (Ser473) and AKT on protein extracts of IL-11RA-silenced A549 and H1975 cell lines. (*C-F*) *In vitro* surrogated assays were performed to analyze tumorigenic properties. (*C*) growth curves, (*D*) colony formation assays (CFA) in 2D, (*E*) transwell migration assays in 3D, and (*F*) 3D soft agar colony formation assay. All experiments were reproduced a minimum of three times in the laboratory and three technical replicates were obtained for each experiment. (*G*-*J*) A549^IL-11RA-KO^ and H1975^IL-11RA-KO^ and control cell lines were injected subcutaneously into athymic nude mice. (*G* and *I*) Relative tumor growth was measured (n=5-6 mice per group). (*H* and *J*) The expression of pSTAT3, cyclin D1 and Ki67 were analyzed by immunohistochemistry, and the quantification of positive cell of pSTAT3, cyclin D1 and Ki67 tumor sections of xenografts are represented. For growth curves, a representative figure is shown. Means and SDs for the technical replicates are shown on the growth curves. The remaining data are representative of at least three independent experiments. Data are given as mean + SD. Statistical significance was determined with a Mann Whitney U-test (**p* <0.05, ***p* <0.01, ****p* <0.001). EV=cell line with empty vector, IL-11RA-KO= silenced IL-11RA variant.


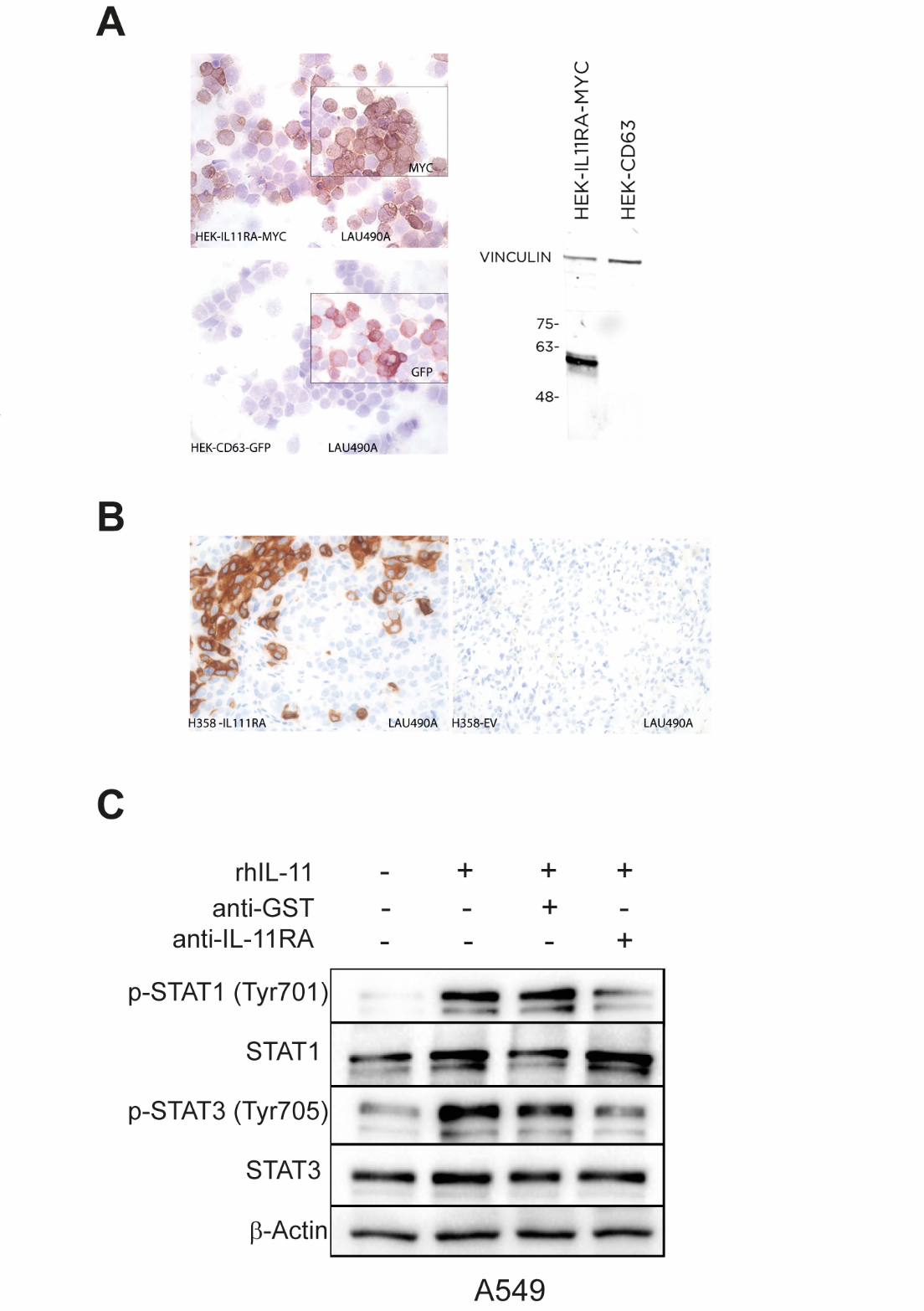


**Figure E5**

**Figure E5 Validation of anti-IL11RA (LAU490A) monoclonal antibody**.

(*A*) Staining of antibody anti-IL11RA (LAU490A) was observed in HEK-IL11RA-Myc transfectants but not in HEK-CD63 (negative control). One band of bands around 50 kDa was detected by western blot (WB) in HEK-IL11RA-MYC cell extracts while no expression was found in HEK-CD63 cells extracts. Anti-vinculin antibody was used as a loading control. (*B*) Immunocytochemical staining for IL11RA on paraffin sections of xenograft H358 over-expressing IL-11RA (16-14533-2) and its control negative, xenograft H358 with the empty vector (16-13529-2). These data confirm the specificity of the neutralizing anti-IL11RA (LAU490A) monoclonal antibody. (*C*) Blockade of IL-11/IL-11RA signaling with the neutralizing anti-IL11RA (LAU490A) monoclonal antibody in A549 cell line pre-treated for 24 h with antibody against IL-11-RA or control mAb (anti-GST) (500 µg/ml). Cells were then stimulated with rhIL-11 (50 ng/ml) for 15 min. After the stimulation, western blot was performed to analyze STAT3 and STAT1 activation. Anti-GST=neutralizing antibody against GST, anti-IL-11-RA=neutralizing antibody against IL-11-RA, rhIL-11=recombinant human IL-11.


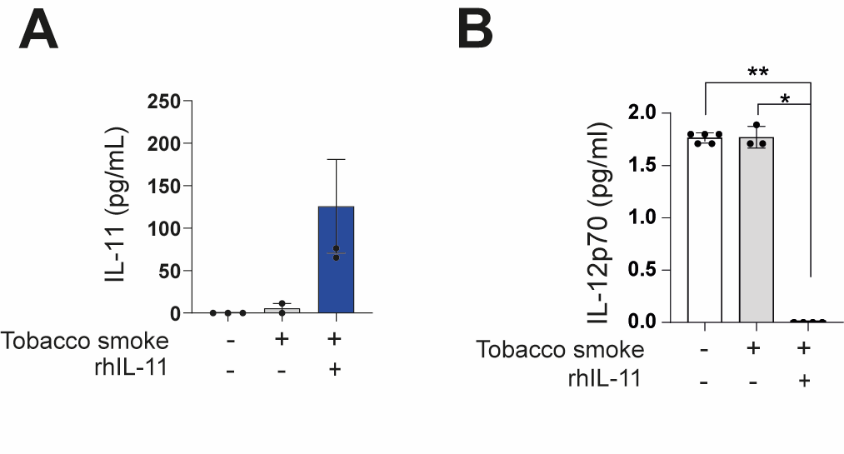


**Figure E6**

**Figure E6 Expression of cytokines in the bronchoalveolar lavage of mice at the end of the tobacco smoke protocol.**

(*A*) Concentrations of human IL-11 from BALFs collected from mice exposed to tobacco smoke (n=2), tobacco smoke plus IL-11 (n=3) and control group (n=3). (*B*) Expression of IL12p70 from BALFs measured by ELISA (air control: n=7; tobacco smoke: n=4; tobacco smoke plus IL-11: n=4).


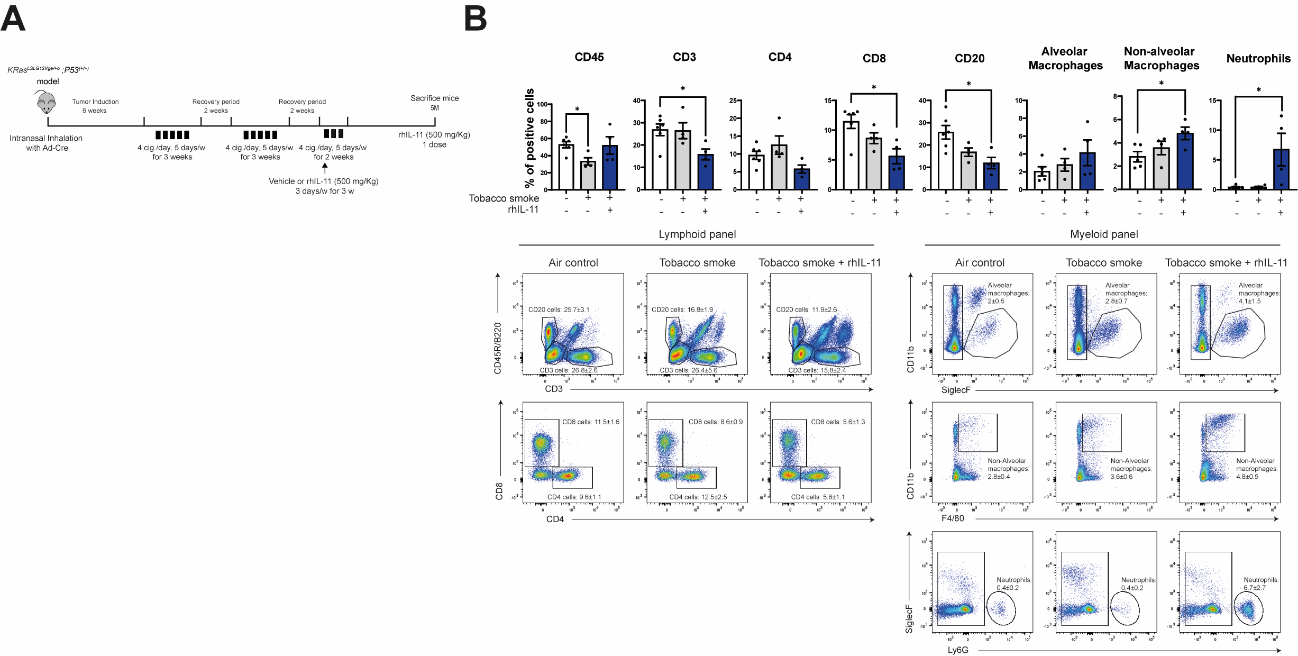


**Figure E7**

**Figure E7 Effect of IL-11 in the immune cell populations in a tobacco-exposure lung cancer mouse model.**

(*A*) Schematic layout of the experiment. in the *KRas^LSLG12Vge/+o^;P53*^(+/+)^ model. After six weeks, mice (n=8) were exposed to TS for three weeks, followed by two week of recovery period. This procedure was repeated twice. In the last cycle, one group of TS-exposure mice were treated with multiple doses of rhI-L11.At five months of age, mice were sacrificed. A third control group of mice (n=6) was exposed to a normal air environment in place of TS. (*B*) Lungs from IL-11 treated mice were analyzed by flow cytometry for the frequencies of infiltrating lymphocytes: CD3 T cells (Live/Dead^-^ CD45^+^ CD3^+^); CD4 T cells (Live/Dead^-^ CD45^+^ CD3^+^ CD4^+^); CD8 T cells (Live/Dead^-^ CD45^+^ CD3^+^ CD8^+^); B cells (Live/Dead^-^ CD45^+^ CD3^-^ CD20^+^); Alveolar macrophages (Live/Dead^-^ CD45^+^ B220^-^ Ly6G^-^ SiglecF^+^); non-alveolar macrophages (Live/Dead^-^ CD45^+^ B220^-^ Ly6G^-^ CD11b^high^ F4/80^+^) and Neutrophils (Live/Dead^-^ CD45^+^ B220^-^ Ly6G^+^). Data are given as mean + SD. Representative dot plots are shown for groups. Statistical significance was determined with the Kruskal-Wallis test** (*p* <0.01), *** (*p* <0.001).


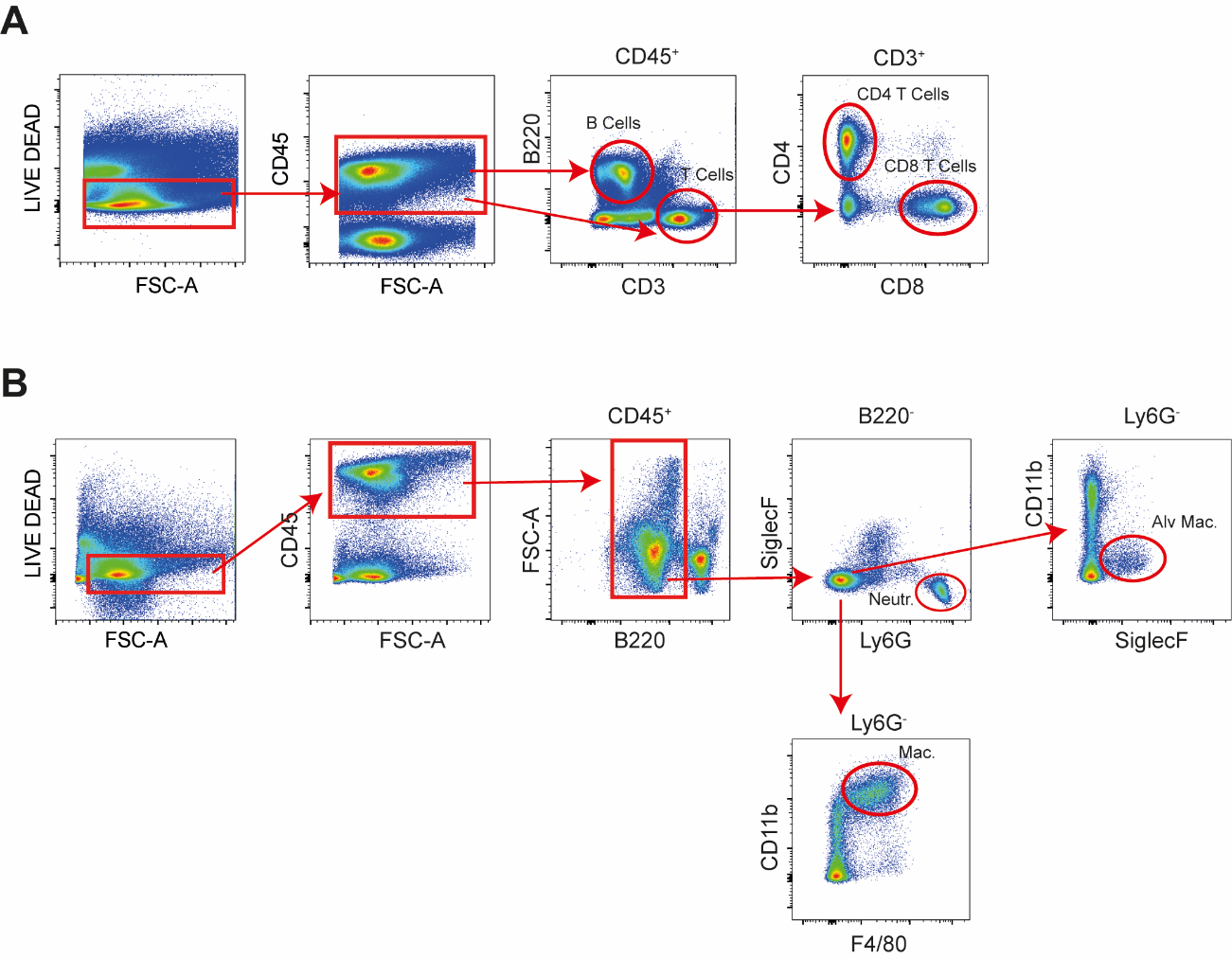


**Figure E8**

**Figure E8 Gating strategy and representative dot plots for immune cell types from tobacco-exposed and IL-11 treated mice.**

In all flow cytometry analyses, we gated live cells that were negative for Live Dead staining and then we selected the immune cell population (CD45^+^ positive cells). (*A*) Gating strategy for lymphoid cell subsets: CD45+, CD3+, B cells, CD4^+^ and CD8^+^ T cells. (*B*) Gating strategy for neutrophils, alveolar macrophages and non-alveolar macrophages. Frequencies of all immune populations were quantified by LSR Fortessa Cytometry.


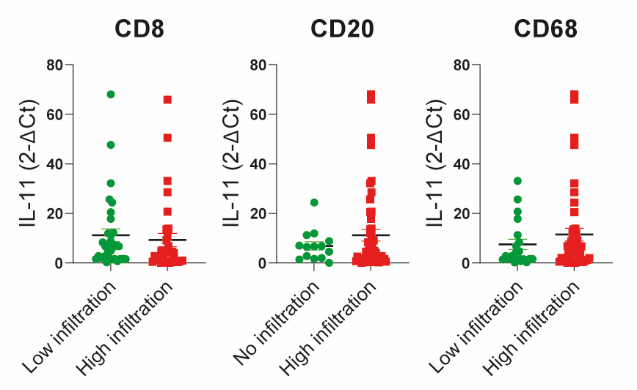


**Figure E9**

**Figure E9 Correlation between IL-11 expression and immune cell populations** Each graph represents the mean expression of IL-11, determined through RT-qPCR, and compared between groups with lower or higher cellular infiltration determined by IHC in the cohort 3. Samples with low CD8 infiltration (N = 32) and high CD8 infiltration (N = 32); samples with null CD20 infiltration (N = 14) and high CD20 infiltration (N = 50); and samples with low CD68 infiltration (N = 20) and high CD68 infiltration (N = 44).

**Supplementary Table E1 Characteristics of the patient cohort from the Hospital Universitario 12 de Octubre used to study the prognostic role of IL-11 (Cohort 1).**

| **Hospital 12 October Cohort** (n=106) | |
| --- | --- |
| **Histology** | Adenocarcinoma |
| **Gender** | Male: 82 (77.4%)  Female: 24 (22.6%) |
| **Median age (years)** | 64.9 [55.3-74.5] |
| **Smoking habit** | Smoker: 40 (37.7%)  Ex-smoker: 39 (36.8%)  Non-smoker: 22 (20.8%)  No data: 5 (4.7%) |
| **Stage** | I: 62 (58.5%)  II: 28 (26.4%)  III: 16 (15.1%) |

Tissue samples were embedded in paraffin and immunohistochemical staining performed. The clinical-pathological characteristics were obtained from the patient's history.

**Supplementary Table E2 Characteristics of the patient cohort from the TCGA used to study the prognostic role of IL-11 (Cohort 2).**

| **TCGA Cohort** (n=527) | |
| --- | --- |
| **Histology** | Adenocarcinoma |
| **Gender** | Male: 244 (46.3%)  Female: 287 (53.7%) |
| **Media age (years)** | 65.4 [55.4-75.4] |
| **Smoking habit** | Smoker: 120 (22.8%)  Ex-smoker: 317 (60.1%)  Non-smoker: 68 (12.9%)  No data: 18 (3.4%) |
| **Stage** | I: 281 (53.3%)  II: 121 (22.9%)  III: 89 (16.9%)  IV: 27 (5.1%)  No data: 9 (1.7%) |

The clinical information and mRNA expression recorded in The Cancer Genome Atlas public database was used in this work.

**Supplementary Table E3 Characteristics of the patient cohort from the Hospital Universitario 12 de Octubre used for transcriptomic studies (Cohort 3).**

| **Hospital 12 October cohort** (n=67) | |
| --- | --- |
| **Histology** | Adenocarcinoma |
| **Gender** | Male: 55 (82.1%)  Female: 12 (17.9%) |
| **Median age (years)** | 65 [62-72] |
| **Smoking habit** | Smoker: 23 (34.3%)  Ex-smoker: 31 (46.3%)  Non-smoker: 9 (13.4%)  No data: 4 (6%) |
| **Stage** | I: 38 (56.7%)  II: 19 (28.4%)  III: 10 (14.9%) |

From the H12O cohort of 106 patients, we used 67 frozen tissue samples and RNA extraction was performed to carry out the transcriptomic analysis.

**Supplementary Table E4 Characteristics of the patient cohort from the Hospital Universitario 12 de Octubre used for digital spatial profile studies (Cohort 4)**.

| **Hospital 12 October cohort** (n=20) | |
| --- | --- |
| **Histology** | Adenocarcinoma |
| **Gender** | Male: 16 (80%)  Female: 4 (20%) |
| **Median age (years)** | 65 |
| **Smoking habit** | Smoker: 7 (35%)  Ex-smoker: 10 (50%)  Non-smoker: 1 (5%)  No data: 2 (10%) |
| **Stage** | I: 10 (50%)  II: 7 (35%)  III: 3 (15%) |

From the H12O cohort of 106 patients, we used 20 FFPE samples for DSP analysis. Clinical-pathological characteristics were obtained from the patient's history.

**Supplementary Table E5 Human Immuno-Oncology panel for protein detection used in the DSP study.**

| **Controls** | **Immune Cell Profiling** | **IO Drug Target** | **Immune Activation Status** |
| --- | --- | --- | --- |
| Rb IgG | PD-1 | 4-1BB | CD127 |
| Ms IgG1 | CD68 | LAG3 | CD25 |
| Ms IgG2a | HLA-DR | OX40L | CD80 |
| Histone H3 | Ki-67 | Tim-3 | ICOS |
| S6 | Beta-2M | VISTA | PD-L2 |
| GAPDH | CD11c | ARG1 | CD40 |
|  | CD20 | B7-H3 | CD44 |
|  | CD3 | IDO1 | CD27 |
|  | CD4 | STING |  |
|  | CD45 | GITR |  |
|  | CD56 |  |  |
|  | CD8 |  |  |
|  | CTLA4 |  |  |
|  | GZMB |  |  |
|  | PD-L1 |  |  |
|  | PanCk |  |  |
|  | SMA |  |  |
|  | Fibronectin |  |  |

**Supplementary Table E6 Antibodies for western blot, flow cytometry and immunohistochemistry analyse employed in this study.**

| **Antibody** | **Technique** | **Reference** |
| --- | --- | --- |
| pSTAT1 (Tyr 701) | Western Blot | Cell Signaling Technology Cat# 9167, RRID:AB_561284 |
| STAT1 | Western Blot | Cell Signaling Technology Cat# 9176, RRID:AB_2240087 |
| pSTAT3 (Tyr 705) | Western Blot/Immunohistochemistry | Cell Signaling Technology Cat# 9145, RRID:AB_2491009 |
| STAT3 | Western Blot | Cell Signaling Technology Cat# 9139, RRID:AB_331757 |
| p-p42/p44 (Trh202/Tyr204) | Western Blot | Cell Signaling Technology Cat# 9101, RRID:AB_331646 |
| p42/p44 | Western Blot | Cell Signaling Technology Cat# 9102, RRID:AB_330744 |
| pAKT (Ser 473) | Western Blot | Cell Signaling Technology Cat# 9271, RRID:AB_329825 |
| AKT | Western Blot | Cell Signaling Technology Cat# 9272, RRID:AB_329827 |
| BCL-2 | Western Blot | Cell Signaling Technology Cat# 15071, RRID:AB_2744528 |
| IL-11RA | Western Blot | Santa Cruz Biotechnology Cat# sc-130920, RRID:AB_212373 |
| p21 | Western Blot | Santa Cruz Biotechnology Cat# sc-6246, RRID:AB_628073 |
| Cyclin D1 | Western Blot | Santa Cruz Biotechnology Cat# sc-450, RRID:AB_627342 |
| β-Actin | Western Blot | Sigma-Aldrich Cat# A5316, RRID:AB_476743 |
| CD45-BV510 clone 30- F11 | Flow Cytometry | BioLegend Cat# 103137, RRID:AB_2561392 |
| B220-PERCPCy5.5 clone RA3/ 6B2 | Flow Cytometry | BioLegend Cat# 103236, RRID:AB_893354 |
| CD3-AF488 clone 17A2 | Flow Cytometry | BioLegend Cat# 100210, RRID:AB_389301 |
| Ly-6G PerCP Cy5.5 clone 1A8 | Flow Cytometry | BioLegend Cat# 127616, RRID:AB_1877271 |
| F4/80 AF647 clone BM8 | Flow Cytometry | BioLegend Cat# 123122, RRID:AB_893480 |
| CD8a-BUV395 clone 53-6.7 | Flow Cytometry | BD Biosciences Cat# 563786, RRID:AB_2732919 |
| B220 PE clone RA3/ 6B2 | Flow Cytometry | BD Biosciences Cat# 553083, RRID:AB_394613 |
| Siglec-F APC-Cy7 clone E50-2440 | Flow Cytometry | BD Biosciences Cat# 565527, RRID:AB_2732831 |
| CD11b BUV395 clone M1/70 | Flow Cytometry | BD Biosciences Cat# 563553, RRID:AB_2738276) |
| CD16/CD32 clone 2.4G2 | Flow Cytometry | BD Biosciences Cat# 553142, RRID:AB_394657 |
| CD4-APC-eFluor780 clone GK1.5 | Flow Cytometry | Thermo Fisher Scientific Cat# 47-0041-82, RRID:AB_11218896 |
| CD45 PEcy7 clone 30-F11 | Flow Cytometry | Thermo Fisher Scientific Cat# 25-0451-81, RRID:AB_2716950 |
| IL-11 | Immunohistochemistry | Thermo Fisher Scientific Cat# PA5-36544, RRID:AB_2553579 |
| IL-11RA | Immunohistochemistry | Abcam Cat# ab125015, RRID:AB_10975018 |
| cyclin D1 | Immunohistochemistry | Agilent Cat# M3642 |
| Ki67 | Immunohistochemistry | Agilent Cat# M7240, RRID:AB_2142367 |
